# Regulation of T cell afferent lymphatic migration by targeting LTβR-mediated non-classical NFκB signaling

**DOI:** 10.1038/s41467-018-05412-0

**Published:** 2018-08-01

**Authors:** Wenji Piao, Yanbao Xiong, Konrad Famulski, C. Colin Brinkman, Lushen Li, Nicholas Toney, Chelsea Wagner, Vikas Saxena, Thomas Simon, Jonathan S. Bromberg

**Affiliations:** 1 Center for Vascular and Inflammatory Diseases, University of Maryland School of Medicine, Baltimore, MD 21201 USA; 2 Department of Surgery, University of Maryland School of Medicine, Baltimore, MD 21201 USA; 3 Department of Laboratory Medicine and Pathology, University of Alberta, 250 Heritage Medical Research Centre, Edmonton, AB T6G 2S2 Canada; 4 Department of Microbiology and Immunology, University of Maryland School of Medicine, Baltimore, MD 21201 USA

**Keywords:** Inflammatory diseases, Tumour-necrosis factors, Lymphatic vessels, NF-kappaB

## Abstract

Lymphotoxin-beta receptor (LTβR) signaling in lymphatic endothelial cells (LEC) regulates leukocyte afferent lymphatic transendothelial migration (TEM). The function of individual signaling pathways for different leukocyte subsets is currently unknown. Here, we show that LTβR signals predominantly via the constitutive and ligand-driven non-classical NIK pathway. Targeting LTβR-NIK by an LTβR-derived decoy peptide (nciLT) suppresses the production of chemokines CCL21 and CXCL12, and enhances the expression of classical NFκB-driven VCAM-1 and integrin β4 to retain T cells on LEC and precludes T cell and dendritic cell TEM. nciLT inhibits contact hypersensitivity (CHS) at both the sensitization and elicitation stages, likely by inhibiting leukocyte migration. By contrast, targeting LTβR-classical NFκB signaling during the elicitation and resolution stages attenuates CHS, possibly by promoting leukocyte egress. These findings demonstrate the importance of LTβR signaling in leukocyte migration and LEC and lymphatic vessel function, and show that antagonist peptides may serve as lead compounds for therapeutic applications.

## Abstract (web summary)

Lymphotoxin beta receptor (LTβR) signalling regulates leukocyte migration through the lymphatic endothelial layers. Here, the authors show that treatment of an LTβR-derived decoy peptide can target the non-classical NFκB pathway to inhibit T cell and dendritic cell migration and ameliorate contact hypersensitivity in mouse models.

## Introduction

Recirculating CD4 T cells enter lymph nodes (LNs) from tissues via afferent lymphatics or from blood across high endothelial venules (HEV). Transendothelial migration (TEM) of the leukocytes from blood to LNs or blood to non-lymphoid tissues has been extensively studied^1^. Alternatively, the regulation of T cell migration from tissues to LNs via afferent lymphatics is far less well defined. Recent studies from this and other laboratories suggest that mechanisms of afferent lymphatic T cell migration are distinct from those used by DC, neutrophil, or monocyte migration, in that T cell migration is governed by integrin-independent mechanisms, such as S1P/S1PR1-mediated homeostatic T cell trafficking^2^ and LTα1β2/lymphotoxin-beta receptor (LTβR)-mediated regulatory T cells (Treg) entry into lymphatics^3^.

The migration of Treg from grafts to LN via afferent lymphatics is critical for graft survival, and cannot be supplanted by Treg migration from blood through HEV into the same LN^4^. Treg specifically employ several molecular mechanisms, distinct from non-Treg CD4^+^ T cells, to migrate through afferent lymphatics^3,5^. A unique mechanism employed by Treg is the high level expression of cell surface lymphotoxin (LT), which is required for migration from the allograft to afferent lymphatics and then to the draining LN^3^. This LT-dependent mechanism is neither required to enter LN via the HEV, nor for egress from the LN to efferent lymphatics. Treg cell surface LT binds to and activates the LTβ receptor (LTβR) expressed on lymphatic endothelial cells (LEC), causing changes in LEC morphology that accompany Treg migration.

**Fig. 1:**
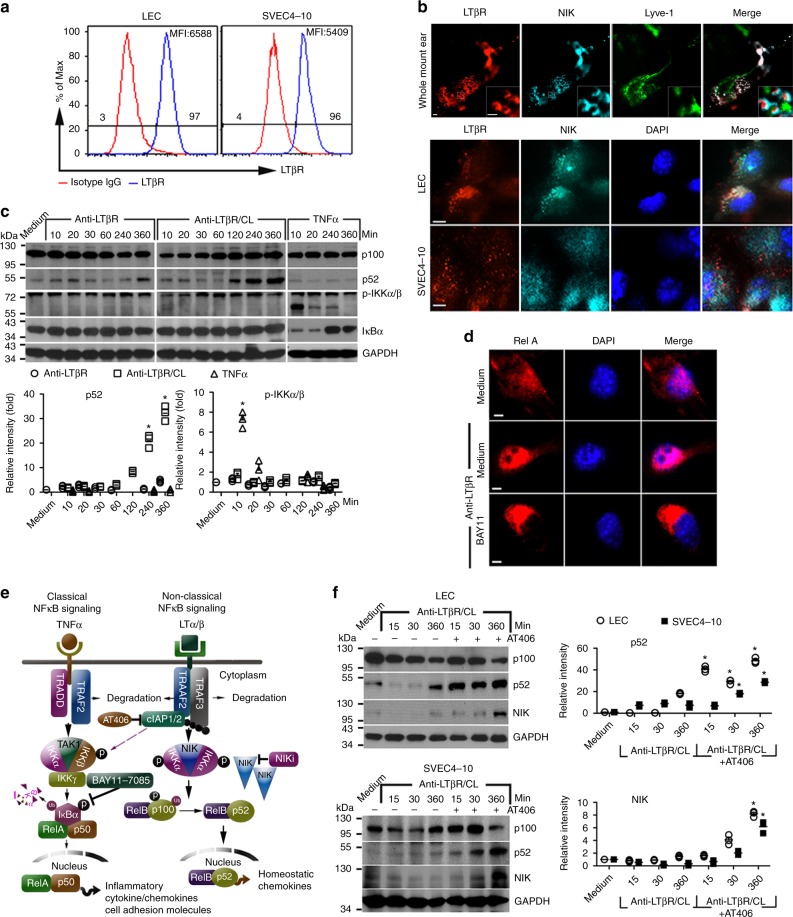
Preferential non-classical NFκB signaling induced by LTβR activation in LEC. **a** Flow cytometry analysis of LTβR expression on murine primary LEC and SVEC4-10. MFI mean fluorescence intensity. **b** Immunohistochemistry of LTβR and NIK expression on mouse whole-mount ear, primary LEC, and SVEC4-10. Magnification ×20, ×60 (inset); scale bar 10 μm. **c** Mouse primary LEC stimulated with 2 μg/mL 3C8 anti-LTβR mAb or 20 ng/mL TNFα for the indicated times. For crosslinking (CL), cells incubated with 2 μg/mL anti-LTβR mAb at 4 °C, washed, and then crosslinked with 2 μg/mL mouse anti-rat IgG1 for the indicated times. Cell lysates immunoblotted for p100, p52, phosphorylated IKKα/β, IκBα, and GAPDH. The bar graphs represent the relative band intensities (mean ± SEM) from three independent experiments. *p *<* 0.05 by one-way ANOVA. **d** Nuclear translocation of RelA/p65 in LEC stimulated with 2 μg/mL anti-LTβR for 10 min. Magnification ×60; scale bar 4 μm. **e** Diagram of TNFα and LTβR-mediated classical and non-classical NFκB signaling. **f** Mouse primary LEC or SVEC4-10 treated as in **c**, along with 25 μM AT406. Cell lysates immunoblotted for p100, p52, NIK, and GAPDH. The bar graphs represent the relative band intensities (mean ± SEM) from three independent experiments. *p *<* 0.05 by one-way ANOVA

LT is a member of the tumor necrosis factor (TNF) superfamily that has major role in lymphoid organogenesis^6,7^. LT has two subunits (LTα and LTβ) and is found in two distinct forms: soluble homotrimer of LTα (LTα3) that binds TNF receptors, and membrane-bound heterotrimer (LTα1β2) that signals via LTβ receptor (LTβR) (shown in Fig. 1e). Unlike TNF receptors (TNFR) that exclusively activate the classical arm of NFκB, LTβR activation induces both the classical and non-classical NFκB pathways^8^. The classical NFκB activation is rapid and transient, involves the Inhibitor of kappa-B kinase (IKK)-complex mediated phosphorylation and degradation of the inhibitor IκBα causing the release of RelA/p50 complex and transfer to the nucleus to allow inflammatory gene transcription. In contrast, activation of non-classical NFκB is gradual and involves NFκB inducing kinase (NIK)-dependent processing of p100 into its transcription-regulatory fragment p52 that dimerizes with RelB and results in nuclear translocation. The recruitment of adaptor protein TNFR-associated factor (TRAF) 2 and 3 to LTβR triggers NFκB signal pathways. For LTβR-mediated p100 processing in LTαβ-activated cells, TRAF2 bridges cellular inhibitors of apoptosis (cIAP) 1/2, an E3 ubiquitin ligase, to degrade TRAF3. TRAF3-deficient cells exhibit constitutive p100 processing. TRAF2 and TRAF3 thus function as mediators and inhibitors of LTβR signaling, respectively^9–11^.

Classical IKKβ-NFκB-dependent genes include inflammatory chemokines (CCL4 and CCL2) and adhesion molecules (VCAM-1, ICAM-1, and ELAM-1). In contrast, alternative IKKα-RelB/p52-dependent genes include homeostatic chemokines involved in lymphoid organogenesis (CCL19, CCL21, and CXCL12)^12,13^. The Treg LTαβ–LEC LTβR interaction favors the non-classical over the classical signaling pathway^3,14^. However, the precise nature of LTβR signaling in LEC and the consequences for LEC structure and function that regulate leukocyte migration remain to be fully defined and quantitated.

LTβRIg, a soluble decoy fusion protein comprised of the ectodomain of LTβR fused to Fc of immunoglobulin G, blocks LT/LIGHT binding to LTβR and has shown efficacy in preclinical disease models as an anti-inflammatory treatment^15^. However, these results have not been consistent across disease models^16,17^. As LTβRIg blocks signaling induced by both LTα1β2 and LIGHT, and blocks both the classical and non-classical NFκB pathways, it is difficult to determine which ligands and signaling pathways are important in the various models. Thus, specific targeting of individual arms of LTβR signaling should reveal the pathway that is responsible for promoting leukocyte trafficking, and enable selective control of leukocyte migration.

Herein we use LTβR-specific cell permeable peptides to selectively target separate arms of the NFκB pathway and reveal the molecular mechanism of leukocyte migration. The results show that constitutive and stimulated afferent LEC LTβR signaling preferentially engage the NIK pathway, resulting in changes in LEC morphology, structure, and gene expression. These NIK-induced effects couple with T cell ligands and receptors to determine TEM into the afferent lymphatics. The results reveal a general requirement for LEC LTβR NIK signaling for normal afferent lymphatic migration of leukocytes. These findings suggest how lymphatic migration may be regulated in homeostasis and during the induction and resolution of inflammatory events.

## Results

### LTβR mediates non-classical NFκB signaling in LEC

Flow cytometry, immunohistochemistry, and western blot analysis of murine primary LEC, the SVEC4-10 LEC line, and lymphatic vessels in vivo confirmed that LEC express high levels of the LTβR (Fig. 1a, b). Although agonist anti-LTβR mAb induced early classical NFκB activation in LEC with weak phosphorylation of IKKα/β or IκBα degradation, TNFα-induced strong IKKα/β phosphorylation and IκBα degradation in the same cells (Fig. 1c), showing that classical and rapid NFκB signaling were intact. Notably, LTβR activation induced nuclear translocation of RelA, which is the essential subunit of classical NFκB (Fig. 1d, e), showing that the classical pathway was indeed induced through LTβR. In contrast, agonist anti-LTβR mAb mediated strong NIK signaling as indicated by increased p100 processing into p52, and p100 processing was further strengthened by crosslinking the agonist antibody (Fig. 1c). AT406, an inhibitor of cIAP1/2, the E3 ubiquitin ligase which targets NIK for proteasome-mediated degradation in resting cells, promoted more LTβR-mediated NIK accumulation and p100 processing in LEC and SVEC4-10 (Fig. 1e, f), further confirming the activation of the NIK signaling pathway by LTβR activation. It was noteworthy that NIK expression was observed in resting primary LEC and SVEC4-10 and in Lyve-1-expressing LEC of mouse ear pinnae in vivo (Fig. 1b), and at baseline there were low levels of p52 present in the LEC and SVEC4-10 (Fig. 1c, f). Furthermore, LTβR and NIK co-localized in LEC confocal images (Fig. 1b). Together, these data suggested that there is low-level constitutive activation of the non-classical pathway in LEC both in vivo and in vitro, and preferential induction of the non-classical pathway by LTβR signaling.

### LTβR induces chemotactic molecules by both pathways

**Fig. 2:**
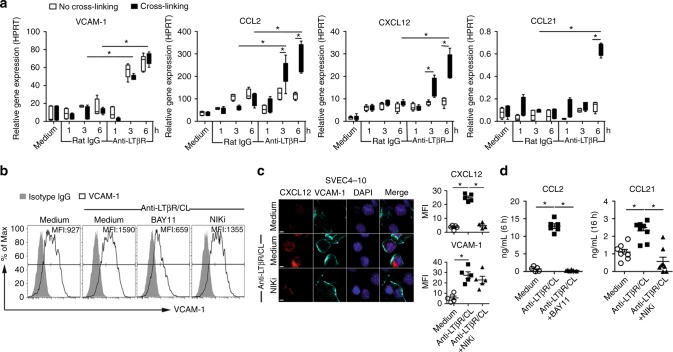
Agonistic anti-LTβR mAb induces inflammatory and homeostatic chemokines and cell adhesion molecules in LEC. **a** qRT-PCR of indicated genes induced by agonistic anti-LTβR mAb or isotype rat IgG crosslinked with or without mouse anti-rat IgG1 in LEC at the indicated times. Treated as in Fig. 1.**b** Flow cytometry analysis of VCAM-1 expression on LEC after treatment with anti-LTβR mAb plus crosslinking, with or without 25 μM NFκB inhibitor BAY11-7085 or 50 μM NIKi for 3 h. **c** LTβR-induced CXCL12 and VCAM-1 expression on SVEC4-10 treated with anti-LTβR mAb plus crosslinking, with or without NIKi (50 μM) for 3 h. Magnification ×60; scale bar 5 μm. **d** Secretion of CCL2 and CCL21 by LEC stimulated with anti-LTβR mAb plus crosslinking, with or without BAY11-7085 (25 μM) or NIKi (50 μM) measured at the indicated times. *p *<* 0.05 by one-way ANOVA (**a**, **d**) or by Student’s *t*-test (**c**)

LTβR ligation on LECs induced VCAM-1 and the inflammatory chemokine CCL2 transcription as early as 3 h after stimulation (Fig. 2a), along with increasing cell surface VCAM-1 (Fig. 2b, c) and CCL2 secretion (Fig. 2d). Crosslinking anti-LTβR mAb on the cell surface to potentiate receptor signaling further enhanced CCL2 transcription (Fig. 2a). LTβR-mediated CCL2 production and VCAM-1 expression were suppressed by BAY11-7085, an irreversible inhibitor of IκBα phosphorylation (Fig. 2b, d). In contrast, the NIK inhibitor 4H-isoquinoline-1, 3-dione (NIKi) did not affect VCAM-1 expression on LEC. These results confirmed the dependence of CCL2 and VCAM-1 expression on the classical NFκB pathway.

Transcription of the homeostatic chemokines CXCL12 and CCL21 gradually peaked 6 h after strong LTβR stimulation induced by crosslinking the agonist mAb (Fig. 2a), and CCL21 secretion was detectable after 16 h (Fig. 2d). Increased intracellular CXCL12 and CCL21 could also be demonstrated after anti-LTβR mAb crosslinking on LEC (Fig. 2a, c, d). CXCL12 and CCL21 production induced by LTβR activation were inhibited by the NIKi (Fig. 2c, d), confirming the non-classical pathway dependence of the expression of these genes. Together, these results showed that LTβR stimulation on LEC resulted in the earlier expression of genes regulated by the classical NFκB pathway followed by the later expression of genes regulated by the non-classical NIK pathway, and suggested differences in LTβR requirements for weak or strong signals.

### LTβR-derived peptides that target separate NFκB pathways

**Fig. 3:**
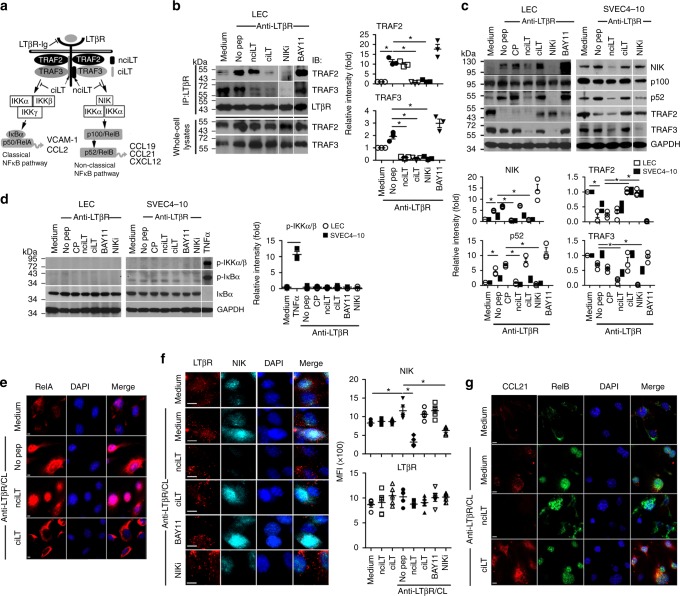
Targeting of LTβR-mediated classical and non-classical NFκB signaling pathways by LTβR-specific peptides. **a** Diagram of peptide selective blockade of separate arms of LTβR signaling. **b** Immunoprecipitation of LTβR complex with anti-LTβR in lysates of LEC pretreated with the indicated peptides (20 μM) and then stimulated with anti-LTβR mAb (2 μg/mL) for 10 min. Complexes run on SDS-PAGE, and immune blotted with anti-TRAF2, anti-TRAF3, and anti-LTβR. **c**, **d** LEC and SVEC4-10 pretreated with indicated peptides (20 μM) or inhibitors (25 μM BAY11-7085; 50 μM NIKi) and then stimulated with anti-LTβR (2 μg/mL) for 6 h (**c**) or 10 min (**d**). In panel **d**, SVEC4-10 stimulated with 20 ng/mL TNFa. Cell lysates immune blotted for p100, p52, NIK, TRAF2, and TRAF3 (**c**); for IKKα/β, and for IκBα phosphorylation and degradation (**d**). **e** Cells treated as in (**d**); immunohistochemistry of RelA. Magnification ×60; scale bar 4 μm. **f**, **g** Cells treated as in (**c**). Immunohistochemistry of LTβR and NIK in SVEC4-10 (**f**); CCL21 and RelB in LEC (**g**). Magnification ×60; scale bar 8 μm (**f**) or 4 μm (**g**). The bar graphs in (**b**–**d**) represent the relative band intensities (mean ± SEM) from three independent experiments. *p *<* 0.05 by one-way ANOVA

LTβR recruits TNF receptor-associated factors (TRAFs) 2, 3, and 5 via its intracellular domain. Mutagenesis studies indicate that TRAF binding to specific motifs in LTβR bifurcates the two arms of NFκB signaling (Fig. 3a)^18,19^. To regulate separate LTβR signaling pathways, we designed decoy peptides comprised of the N-terminal cell-penetrating sequence of the *Drosophila* antennapedia peptide (RQIKIWFQNRRMKWKK) plus one of the TRAF-binding motifs in LTβR to specifically target each arm of the NFκB pathway (Fig. 3a). nciLT(RQIKIWFQNRRMKWKKTGNIYIYNGPVL) harbored the sequence required for TRAF2 and TRAF3 recruitment into the activated, non-classical LTβR complex and p100 processing^18^. ciLT (RQIKIWFQNRRMKWKKTPEEGAPGP) included the (P/S/A/T)X(Q/E)E TRAF-binding motif required for TRAF2 but not TRAF3 binding to LTβR in the classical pathway^18,20^. A control peptide (RQIKIWFQNRRMKWKKGEHGQVAHGA) included the random sequence of LTβR amino acids. The effective doses and incubation periods for the peptides were determined by cytokine (CCL2, CCL21, CXCL12) and receptor (VCAM-1) mRNA expression responses of SVEC4-10 maximally activated by crosslinking of agonist anti-LTβR mAb and treated with various doses of nciLT and ciLT (Supplementary Fig.1). The results showed that a concentration of 20 μM of each peptide gave optimal results, similar to our previous experience with peptides of different specificities^21,22^.

To evaluate their blocking activities in signaling responses, LEC were incubated with these blocking peptides prior to activation with anti-LTβR mAb, and compared to conventional, receptor nonselective inhibitors of the separate NFκB signaling pathways. With respect to classical signaling in LEC and SVEC4-10 cells, TRAF3 constitutively bound to the LTβR, and LTβR activation induced rapid TRAF2 recruitment to the receptor after 10 min (Fig. 3b) followed by later TRAF2 degradation after 6 h (Fig. 3c). Immunoprecipitation showed that ciLT but not BAY11-7085 was sufficient to inhibit both TRAF2 and TRAF3 precipitating with the LTβR complex (Fig. 3b), either by preventing binding to and/or inducing dissociation from the receptor. ciLT but not BAY11-7085 also prevented TRAF2 degradation (Fig. 3c). These results indicated that ciLT acted as an inhibitor of the LTβR upstream classical NFκB pathway, while BAY11-7085 acted as a downstream inhibitor of the classical NFκB receptor/TRAF complex. In keeping with the low level of classical NFκB activities induced by the LTβR in these cells (Fig. 1c), there was no observed LTβR-induced rapid phosphorylation of IKKα/β or IκB, or IκB degradation in LEC and SVEC4-10 (Fig. 3d). Nonetheless, nuclear translocation of RelA and early response genes of the classical pathway (CCL2, VCAM-1) were induced by LTβR activation and the translocation and gene expression were specifically inhibited by ciLT (Fig. 3e, Supplementary Fig.1).

With respect to the non-classical pathway, activation of LTβR in LEC and SVEC4-10 induced rapid and strong NIK accumulation followed by p100 processing to p52 (Fig. 3c, f). Notably, there was TRAF3 binding to LTβR in unstimulated LECs (Fig. 3b) suggesting, as noted above (Fig. 1b, c, e), low level constitutive activation of the non-classical pathway via the LTβR. Immunoprecipitation showed that nciLT blocked TRAF3 but not TRAF2 binding to the LTβR complex (Fig. 3b) and prevented p100 processing to p52 (Fig. 3c). In comparison, NIKi blocked both TRAF2 and TRAF3 recruitment to the receptor, prevented downstream p100 processing to p52, and inhibited TRAF2 degradation. These results showed that nciLT specifically sequestered TRAF3 from the receptor complex, and did not prevent TRAF2 degradation, but did prevent NIK accumulation for non-classical NFκB signaling. In contrast, NIKi prevented TRAF2 degradation, which is required for classical NFκB activation, suggesting nciLT was a more specific NIK pathway inhibitor than NIKi. In fact, NIKi had a partial inhibitory effect on CCL2 transcription activated by the classical pathway (Supplementary Fig. 2). Late response genes of the non-classical pathway (CCL21, CXCL12) were induced by LTβR activation and their expression was specifically inhibited by nciLT (Supplementary Fig. 1).

Immunohistochemistry showed co-expression of LTβR and NIK on LEC and SVEC4-10, with co-localization of the two molecules in confocal images (Figs. 1b, 3f). nciLT and NIKi inhibited NIK expression and its co-localization with LTβR, without affecting LTβR expression (Fig. 3f). In contrast, neither ciLT nor BAY11-7085 affected NIK expression or co-localization with LTβR. In addition, LTβR activation induced RelB nuclear translocation which was suppressed by nciLT but not ciLT (Fig. 3g). Together, these data confirmed the specificity and mechanism of activity of nciLT in comparison to the other three molecules.

### Gene profiles are regulated by LTβR-NFκB-blocking peptides

**Fig. 4:**
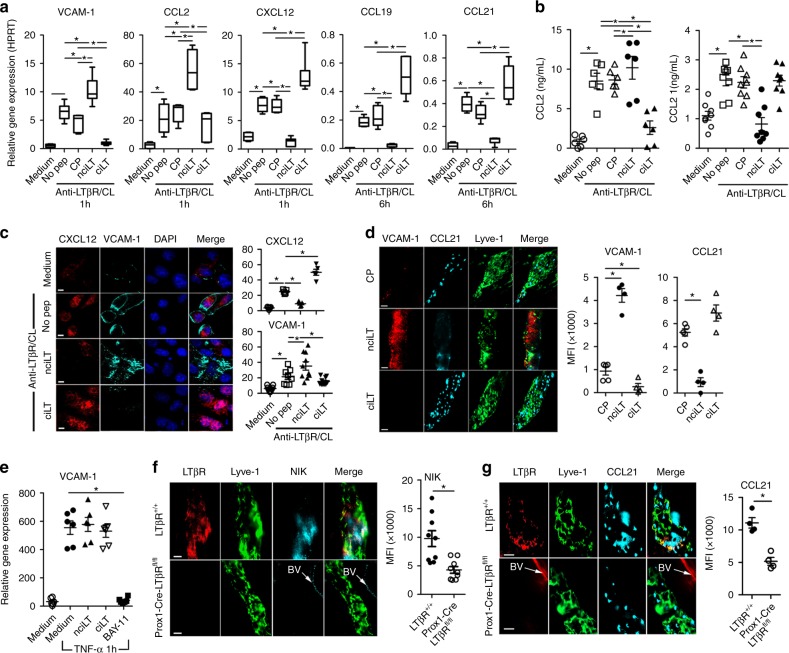
Regulation of gene profiles by the LTβR NFκB-blocking peptides. **a** qRT-PCR of VCAM-1 and chemokines in LEC stimulated with anti-LTβR mAb (2 μg/mL) plus CL with mouse anti-rat IgG1 (2 μg/mL) for the indicated times. **b** ELISA for CCL2 and CCL21 in the LEC supernatants after 4 or 16 h stimulation as in **a**, respectively. **c** CXCL12 and VCAM-1 expression in LEC after 3 h stimulation as in **a**. Magnification ×40; scale bar 20 μm. **d** C57BL/6 mice injected with 5 nmol/ear control peptide (CP), nciLT or ciLT; after 3 h ears were fixed for whole-mount ear staining. Magnification ×20; scale bar 50 μm. **e** qRT-PCR of VCAM-1 in LEC stimulated with TNFα (20 ng/mL) for 1 h. **f**, **g** Whole-mount ear staining of NIK and CCL21 in Prox1-Cre-ER^T2+/−^LTβR^fl/fl^ mice 10 days after tamoxifen treatment (0.125 mg/g i.p. for 5 consecutive days). BV blood vessel. Magnification ×60; scale bar 10 μm. *p < 0.05 by one-way ANOVA (**a**–**e**) or by Student’s *t-*test (**f**, **g**)

To further validate the peptide inhibitor specificities and mechanisms, their actions were related to the separate LTβR signaling pathways and specific gene activation. The blocking peptides were assessed for their effects on early classical pathway and later non-classical pathway gene activation and expression in LEC. nciLT, which blocked LTβR-non-classical NFκB signaling, enhanced early classical pathway CCL2 and VCAM-1 expression and inhibited homeostatic CXCL12, CCL19, and CCL21 chemokine production via the non-classical pathway, as measured by RT-PCR (Fig. 4a), ELISA (Fig. 4b), and immunohistochemistry (Fig. 4c). nciLT was also effective in vivo and significantly decreased the expression of CCL21 and increased VCAM-1 expression on Lyve-1^+^ lymphatic vessels (Fig. 4d). Conversely, ciLT blockade of the LTβR-classical NFκB pathway suppressed classical pathway induced CCL2 and VCAM-1 and enhanced non-classical homeostatic chemokines CXCL12, CCL19, and CCL21 (Fig. 4a–d). Both peptides showed LTβR-specific inhibition since TNFR1-mediated VCAM-1 expression was not affected by either nciLT or ciLT, yet was significantly inhibited by BAY11-7085 (Fig. 4e). Thus, these peptides specifically inhibited gene expression regulated by the classical and non-classical pathways engaged by LTβR. Selective deletion of LTβR in Prox1-expressing lymphatics 10 days after tamoxifen treatment of Prox1-Cre-ER^T2+/−^LTβR^fl/fl^ mice also diminished NIK and CCL21 expression (Fig. 4f, g), confirming the importance of LTβR and NIK signaling.

### Targeting non-classical signaling inhibits T cell migration

**Fig. 5:**
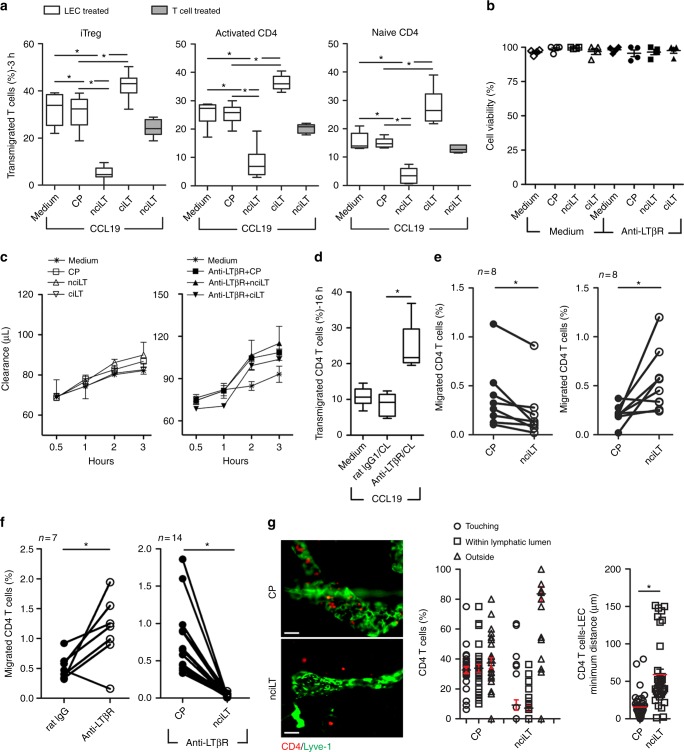
LTβR peptides that block non-classical NFκB signaling pathway inhibit CD4 T cell migration across LEC. **a** LEC cell layers pretreated with the indicated peptides (20 μM) for 30 min at 37 °C and then Foxp3^+^CD4^+^CD25^+^ iTregs, Foxp3^−^CD25^−^CD4^+^aCD4, or naïve CD4 cells loaded into upper chamber and migrated toward CCL19 (50 ng/mL) for 3 h. **b** LEC incubated with 20 μM nciLT or ciLT peptides for 3 h without or with stimulation with 2 μg/mL anti-LTβR mAb. Cell viability determined by MTT incorporation assay. **c** Time-course monitoring of 0.75% albumin/Evans Blue diffusion across LEC cell layer in Boyden chamber treated as in **b**. **d** LEC cell layers pretreated with anti-LTβR mAb or isotype rat IgG for 30 min at 4 °C, washed and CL with mouse anti-rat IgG1 for 30 min at 37 °C, followed by 16 h incubation, then naïve CD4 cells loaded into the upper chambers and migrated toward CCL19 (50 ng/mL) for 3 h. **e**, **f** Footpad-popliteal LN migration assay. 1 × 10^6^ CFSE-labelled naïve CD4 T cells mixed with 5 nmol CP (left side), 5 nmol nciLT (right side), or 5 nmol ciLT (right side) without (**e**) or with 1 μg anti-LTβR mAb (**f**), injected into hind footpads. After 16 h popliteal LNs collected and analyzed with flow cytometry. **g** Ear whole-mount staining and migration. CFSE-naïve CD4 T cells injected into ear pinnae pretreated with peptides (5 nmol/ear), and collected after 16 h post injection. Images of T cells and Lyve-1^+^ lymphatic vessels (left). Magnification ×20; scale bar 50 μm. Position (center) and distance (right) of T cells with respect to lymphatic vessels. **a**–**g** Mean ± SEM of at least three independent experiments. *p < 0.05 by one-way ANOVA (**a**–**d**) or by Student’s *t-*test (**e**–**g**)

We previously demonstrated that CD4^+^ Treg but not CD4^+^ non-Treg utilized cell surface LTαβ to engage LTβR on LEC to migrate across the afferent lymphatic endothelium in vitro and in vivo. Treg expressed higher levels of LTαβ than non-Treg, and modulated membrane and cytoskeletal structure of LECs via LTβR-mediated NIK signaling^3^. To further investigate the roles of the separate signaling pathways, we evaluated the functional efficacy of the blocking peptides on Treg migration. In vitro transmigration assays showed that when LEC but not Treg were pretreated with blocking peptides, that nciLT prevented Treg TEM across LEC (Fig. 5a, left panel). In contrast, pretreatment of LEC with ciLT, the classical pathway blocking peptide, enhanced Treg migration, likely by modulating the expression of migration molecules regulated by the non-classical pathway, such as increased CCL21 and CXCL12 expression (Fig. 4a–d). The peptides did not affect LEC viability or permeability as shown by the MTT viability and Evans Blue barrier assays, respectively (Fig. 5b, c). Thus, migration inhibition was not due to non-specific effects on LEC viability or permeability. When LEC were incubated with peptides and then washed, the effect of the peptides persisted for more than 6 h prior to the migration assay, and were reversed by 12–16 h (Supplementary Fig. 3a).

nciLT also inhibited TEM of activated and naive CD4^+^ non-Treg T cells across LEC (Fig. 5a, middle and right panels). Our previous work demonstrated that additional engagement and activation of LTβR was required for TEM of Treg but not other T cells. The biochemical and immunohistochemical analyses showed that there was constitutive LTβR-NIK expression and activation in LEC, with TRAF3 occupancy (Fig. 3b, c) along with low levels of p52 (Fig. 1f). The current results showed that other T cells required the constitutive activation of LEC LTβR and LTβR-NIK signaling for TEM. Thus, nciLT likely inhibited constitutive receptor activity required for all CD4 T cell TEM. When LEC were specifically activated for 16 h with anti-LTβR mAb plus crosslinking, this resulted in increased migration of naïve CD4 T cells (Fig. 5d), showing that receptor signaling and migration could be enhanced beyond constitutive receptor activation.

We observed similar effects on T cell migration in vivo. Injection of nciLT into the footpad inhibited naïve CD4 T cell homing into local draining LNs, while ciLT injection increased the migration (Fig. 5e). Injection of stimulatory anti-LTβR mAb into the footpad enhanced migration, and this enhanced migration was blocked by nciLT (Fig. 5f). Injection of nciLT also inhibited CD4 T cells from entering into the lymphatic vessels in the ear pinna assay (Fig. 5g). These results confirmed the role for LTβR signaling and the NIK pathway for afferent lymphatic migration in vivo in both basal and stimulated conditions. Once injected into the footpads, the effects of the peptides persisted more than 20 h prior to the migration assay, and were reversed by 36 h (Supplementary Fig. 3b).

### NIK inhibition enhances T cell binding to LEC

**Fig. 6:**
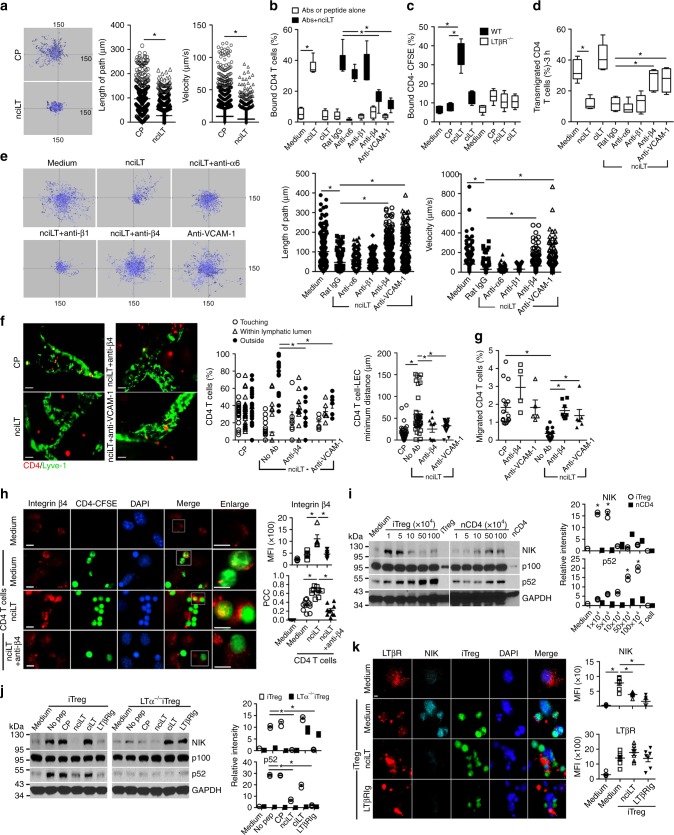
Inhibition of T cell stimulated LTαβ–LTβR NIK pathway on LEC enhances T cell binding to LEC through integrin β4 and VCAM-1. **a** Time-lapse, 2-D microscopy of CD4 T cell migration across LEC. LEC layers in Boyden chambers treated with 20 μM CP or nciLT for 30 min at 37 °C, washed and naïve CD4-CFSE T cell migration to CCL19 (50 ng/mL) monitored for 3 h by live imaging. **b** Binding of naïve CD4 T cells to LEC pretreated with the indicated peptides (20 μM) with or without anti-integrin mAbs (2 μg/mL) or anti-VCAM-1 mAb (3 μg/mL) for 30 min at 37 °C. **c** Binding of naïve CD4 T cells to wild type (WT) or LTβR^−/−^ LEC pretreated with the indicated peptides (20 μM). **d** LEC layers in Boyden chamber treated as in **b**, then naïve CD4 T cells migrated across LEC. **e** LEC layers treated with peptides and mAbs as in **b** for 30 min at 37 °C, washed, and naïve CD4 T cell migration toward CCL19 (50 ng/mL) monitored for 3 h of live imaging. **f** Ear whole-mount staining and migration. Pinnae injected with nciLT (5 nmol/ear) with or without anti-integrin β4 (5 μg) or anti-VCAM-1 mAbs (15 μg) 30 min before transfer of naïve CSFE-labeled CD4 T cells (1 × 10^6^/ear). After 16 h, ears collected and stained for Lyve-1. Images of T cells and Lyve-1^+^ lymphatic vessels (left). Magnification ×20; scale bar 50 μm. Position (center) and distance (right) of T cells with respect to lymphatic vessels. **g** Footpad-popliteal LN migration assay. Hind footpads injected with the same doses of peptide or antibodies as in **f**, then 1 × 10^6^ naïve CD4-CFSE T cells transferred. After 16 h, popliteal LN collected and analyzed with flow cytometry. **h** Expression of cell surface integrins on primary LEC. LEC treated with 20 μM nciLT with or without anti-integrin β4 mAb (2 μg/mL) prior to co-culture with 2 × 10^5^ naïve CD4-CFSE for 3 h. Unbound T cells gently washed away before fixation. PCC Pearson’s correlation coefficient. Magnification ×60; scale bar 5 μm. **i**, **j** Immunoblot of NIK activation in LEC pretreated with the indicated peptides (20 μM) or LTβRIg (2 μg/mL) for 30 min, then incubated with various numbers of purified iTreg or naïve CD4 T cells (**i**) or 5 × 10^5^ wild type or LTα-deficient iTreg (**j**) for 6 h. The bar graphs represent the relative band intensities (mean ± SEM) from three independent experiments. **k** Expression of LTβR and NIK in primary LEC pretreated with 20 μM nciLT or 2 mg/mL LTβRIg for 30 min at 37 °C, and co-cultured with 2 × 10^5^ iTreg-CFSE for 6 h. Magnification ×60; scale bar 5 μm. *p < 0.05 by Students’ *t-*test (**a**) or by one-way ANOVA (**b**–**k**)

We also noted that treatment of LEC with nciLT resulted in markedly increased firm attachment of the LEC to the culture surfaces compared to untreated, control peptide treated, or ciLT-treated LEC. This was true both for resting LEC and LEC in which the LTβR was stimulated with the agonist mAb, again showing that LTβR-NIK constitutive and induced signaling were important for LEC layer structure. Given the observation of increased LEC adhesion, and the decreased T cell migration noted above (Fig. 5a, e, f), we questioned if there were also alterations in T cell adhesion to LEC. Live 2-D migration imaging showed that the CD4 T cells were tethered on nciLT-treated LEC, with significantly lower motility as measured by pathway length and velocity compared to CD4 T cells on control peptide-treated LEC (Fig. 6a). Decreased mobility was accompanied by increased binding of CD4 T cells to nciLT but not ciLT or control peptide-treated LEC (Fig. 6b). In contrast, nciLT did not increase the binding of CD4 T cells to LTβR^−/−^ LEC (Fig. 6c), further confirming the role of LTβR in T cell–LEC adhesion.

Since T cells bind endothelial cells through a variety of adhesion molecules, blocking antibodies to several of these receptors^23,24^ were tested for their activity in the binding, migration, and motility assays. Notably, the enhanced binding of CD4 T cells was diminished when the nciLT-treated LEC were pre-incubated with anti-integrin β4 or anti-VCAM-1, but not a variety of other antibodies (Fig. 6b). Concordantly, CD4 T cell migration across the nciLT-treated LECs, and CD4 T cell motility on nciLT-treated LECs, were restored by these antibodies, but not antibodies to a variety of other integrins (Fig. 6d, e, Supplementary Fig. 4a–c). As noted in Fig. 5e–g, nciLT inhibited migration into lymphatic vessels and the draining LN in vivo. Anti-integrin β4 and anti-VCAM-1 also restored these in vivo migration events (Fig. 6f, g). The importance of VCAM-1 for leukocyte TEM across lymphatic endothelium has previously been reported by others and us^3,25^. Integrin β4 is involved in endothelial cell integrity^26^. Unlike other integrin β chains which can bind multiple α chain partners, the β4 chain only pairs with the α6 chain, while integrin α6 also pairs with β1 (ref. ^27^). The anti-β4 mAb used here (346-11A) recognizes the ectodomain of integrin β4, and has been suggested to dissociate the α6β4 complex^27^. The anti-α6 mAb used here (GoH3) inhibits integrin binding to laminin, but has not previously been shown to alter cell migration or motility. The ability of anti-β4 and the failure of anti-α6 mAb to alter T-LEC interactions here is likely due to the differences in subunit pairing and in the precise epitope specificity of the mAbs.

To investigate how nciLT altered LEC adhesion receptors and thereby T cell movements, we examined surface expression and distribution. As noted above (Figs. 2a–c, 4a, c–e, Supplementary Fig. 2a), LTβR signaling and nciLT each caused a significant increase in the expression of VCAM-1 on LEC and SVEC4-10 via stimulation of classical NFκB signaling. Flow cytometric and immunohistochemical analysis of primary LEC in vitro and Lyve-1-expressing lymphatic vessels in vivo demonstrated the co-expression of integrins α6 and β4, but not integrins αE or β7 (Supplementary Fig. 4d–f). nciLT slightly increased the expression of integrins α6 and increased β4 to a much greater degree on LEC in vitro. Next, naïve CD4 T cells were incubated with primary LEC, unbound T cells washed after 3 h, and the intercellular integrin interactions were examined. Since CD4 T cells did not express integrin β4, but did express β7 and α6 (Supplementary Fig. 4f), the β4 staining was specific for the LEC. CD4 T cells induced a small increased in integrin β4 expression along with co-localization of T cells with β4 expressing LEC under control conditions (Fig. 6h, second row). nciLT again resulted in a marked increase in integrin β4 expression and markedly more co-localization of the T cells and integrin β4 on the LEC (Pearson correlation coefficient (PCC): 0.64 ± 0.1), suggesting clustering of the integrin at the T cell–LEC interface (Fig. 6h, third row). This T cell-LEC interaction was inhibited by anti-integrin β4 mAb (PCC: 0.22 ± 0.1) (Fig. 6h, lower row), commensurate with the binding and motility assays (Fig. 6a–g). Overall, these results demonstrated that LTβR-NIK regulated LEC integrin β4 and VCAM-1-dependent adhesion, motility, and migration of CD4 T cells by altering the expression and distribution of these molecules.

### T lymphocytes activate LEC via the LTαβ–LTβR NIK pathway

We recently demonstrated that Treg but not non-Treg use LTαβ to engage the LTβR–NIK pathway on afferent LEC for TEM to the dLNs^3^. We further investigated the regulation of the LTβR–NIK pathway on LEC and the consequences for T cell migration. Compared to naïve or activated CD4 T cells, iTregs specifically expressed higher levels of cell surface LTαβ (Supplementary Fig. 5a, b)^3^. Primary LECs were incubated with various doses of iTregs or CD4 T cells, and NIK activation was evaluated by immunoblot of LEC lysates after the T cells were removed. Consistent with LTβR expression levels, iTreg induced significantly higher levels of NIK activation than naïve or activated CD4 T cells, while LTα-deficient Treg failed to induce NIK activity (Fig. 6i, j, Supplementary Fig. 5c). Moreover, nciLT but not ciLT suppressed iTreg-induced NIK accumulation and p100 processing, and LTβRIg also blocked NIK activation (Fig. 6j). Incubation of iTreg with LEC resulted in concentration of LTβR at sites of Treg-LEC contact and the induction of NIK and CCL21, and these events were suppressed by nciLT, while VCAM-1 expression was increased (Fig. 6k, Supplementary Figs. 4c, 5d). In contrast, neither naïve nor activated CD4 T cells induced CCL21, while activated CD4 T cells induced a minor increase in VCAM-1 (Supplementary Figs. 4d, 5e, f). Together, these results demonstrated direct stimulation of LEC LTβR by T cell LTαβ, preferential stimulation of the NIK pathway by this interaction, and markedly higher activity from Treg due to higher expression of LTαβ.

### Targeting LTβR alters contact hypersensitivity

**Fig. 7:**
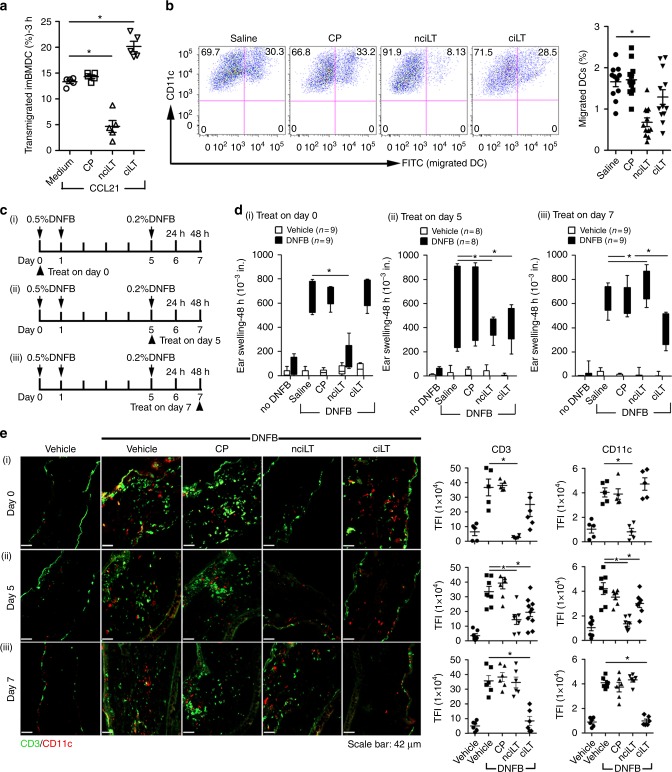
Inhibition of LTβR signaling alters CHS and leukocyte migration. **a** In vitro DC migration to CCL21 (50 ng/mL) through LEC treated with the indicated peptides (20 μM). **b** In vivo DC migration assay. i.d. injection of the indicated peptides (20 nmol) to the shaved abdomen, followed 30 min later by FITC painting. After 16 h, inguinal and axillary LNs collected and analyzed with flow cytometry. **c** Experimental design schematic. 20 nmol of indicated peptides injected i.d. in the abdomen (100 μL, day 0) or 10 nmol in each ear (30 μL, day 5 or day 7) 30 min before DNFB painting on days 0 or 5. **d**, **e** CHS analyzed by ear swelling measured 48 h after DNFB challenge (**d**), or immunohistochemistry for CD11c^+^ DC and CD3^+^ T cells (**e**). Magnification ×20; scale bar 42 μm. TFI total fluorescence intensity. Representative of three independent experiments with 3 mice/group. *p < 0.05 by one-way ANOVA

Contact hypersensitivity (CHS) is a T cell-mediated inflammatory response. Hapten sensitization induces migration of dermal DC (dDC) to dLNs for T cell priming^28,29^. Hapten challenge induces another round of dDC migration from skin to dLNs, stimulation of primed T cells, and recruitment of activated T cells to the site of challenge^30^. During resolution of inflammation, egress of the inflammatory cells out of the CHS site is required^31^. To test the ability of selective NF-κB-blocking peptides to also regulate DC migration, we determined that as for T cells, DC TEM in vitro was inhibited by nciLT and enhanced by ciLT (Fig. 7a). nciLT also inhibited skin Langerhans DC migration from skin to dLN in vivo in response to skin painting with FITC (Fig. 7b).

We next assessed the peptides in CHS to provide proof of concept for manipulation of LEC LTβR signaling in an in vivo immunity model. Our results and the literature show that: (1) the peptides specifically affect T cell and DC migration from tissues to draining LN (results here); (2) the LTαβ–LTβR interaction does not affect T cell migration from blood to tissues, blood to LN, or LN to efferent lymphatics^3^; (3) the LTαβ–LTβR interaction does not affect T cell viability, proliferation, or apoptosis^3^(and results here); (4) for LTα KO and LTβR KO mice there are no changes in migration of T cells to spleen or tissues (note that those mice lack LNs)^3,6,7^; (5) DC express LTαβ^32–34^ and LTβR^35^, which have been implicated in DC homeostasis but not antigen presentation; and (6) the Drosophila Antennapedia based peptides, designed to bind and rapidly enter cells, unlike typical peptide antigens^21,22^ are not substantially delivered to draining LNs. The peptides injected in the footpad did not change draining LN architecture, cell distribution, or cell content from 6 to 24 h post injection. Nevertheless, it was possible that the peptides did not affect residential but rather recirculating leukocyte afferent migration (Supplementary Fig. 53c, d).

To test the in vivo ability of selective NFκB-blocking peptides to regulate the stages of the CHS response, we administered the peptides at the time of DNFB sensitization (day 0), challenge (day 5, elicitation phase), or 48 h after challenge (day 7, resolution phase) (Fig. 7c). Administration of nciLT to the shaved abdomen by i.d. injection 30 min before sensitization, inhibited CHS 24 h after challenge, with fewer T cells and DC infiltrating the ear compared with controls (Fig. 7di, ei, Supplementary Fig. 6i). Inhibition by nciLT of dDC migration to the dLNs likely may have reduced T cell priming. Additionally, CCL21 production by LEC has a critical role for DC afferent lymphatic migration in CHS^28,36^, and was suppressed by nciLT (Fig. 4d). Treatment with nciLT or with ciLT in the ear pinna at the time of DNFB challenge and elicitation on day 5 inhibited CHS with reduced T cell infiltration (Fig. 7dii, eii, Supplementary Fig. 6ii). nciLT at this stage may have again interfered with dDC migration to the dLN and stimulation of primed T cells. ciLT may have blocked expression of classical NFκB-driven inflammatory chemokine (e.g., CCL2) and adhesion molecules (e.g., VCAM-1, ICAM-1) by LEC, which are upregulated and important during CHS^37–39^. ciLT may have also enhanced egress of inflammatory cells out of the ear. By administration of the peptides on day 7, at the start of the resolution stage, nciLT sustained CHS, likely possibly by preventing egress of the inflammatory infiltrate as shown by enhanced T cell and DC infiltration in the ear (Fig. 7eiii). In contrast, ciLT enhanced resolution (Fig. 7diii, Supplementary Fig. 6iii), possibly by inhibiting cytokines and promoting the egress of the inflammatory cells, as fewer CD3 and CD11c cells were present (Fig. 7eiii).

## Discussion

Here we found LEC and lymphatic vessels expressed high levels of LTβR which signaled to both classical and non-classical NFκB pathways^12,13,40^. NIK, the hallmark of non-classical NFκB signaling, was constitutively active in resting LEC, and signaling was enhanced by LTβR activation. LTβR NIK stimulation induced RelB nuclear translocation, chemokines such as CCL21 and CXCL12 which promoted T cell or DC migration across LEC, and enhanced T cell TEM migration. LTβR activation also resulted in classical NFκB signaling, rapid RelA nuclear translocation, and VCAM-1 and CCL2 expression associated with cell migration during inflammation. VCAM-1 expression on LEC or lymphatic vessels was enhanced by the non-classical NFκB inhibitor nciLT, which caused firm adhesion of T cells on LEC, further showing that non-classical and classical NFκB signaling interacted to regulated LEC function. In contrast, the classical NFκB-blocking peptide ciLT enhanced CCL21 production in LEC and promoted T cell migration. Molecular interactions indicated upstream crosstalk between the two arms of LTβR–NFκB pathways, such that nciLT or ciLT decoy peptides influenced TRAF3 or TRAF2 recruitment to the receptor. Together these data demonstrated major roles for LTβR related NFκB pathways in regulating LEC function and TEM.

TRAFs play distinct functional roles for different receptors and cell types. Differential recruitment of TRAF2 and TRAF3 to the LTβR contributes to distinct signaling functions^18,41^. Our results suggested that in primary resting LEC, TRAF3 constitutively bound LTβR resulting in basal low level NIK activity (Supplementary Fig. 7ai). TRAF3 does not directly ubiquinate NIK, but rather bridges the TRAF2-cIAP1/2 complex to NIK, leading to rapid turnover of NIK in resting cells^41,42^. Thus, the preferential binding of TRAF3 instead of TRAF2 to the receptor may favor constitutive NIK activation. Inhibition of cIAP1/2 resulted in an increase in NIK and p100, confirming the direct involvement of cIAP1/2 in NIK decay. After receptor activation, TRAF2 was recruited to the receptor complex, in which cIAP1/2 ubiquinates and degrades TRAF2 and TRAF3 (Supplementary Fig. 7aii). Once TRAF2 and TRAF3 are degraded, NIK is released from the complex and stabilized from degradation by cIAP1/2. Sequestration of TRAF3 from the receptor complex by the decoy receptor nciLT targeted TRAFs and NIK for ubiquitination by cIAP1/2, thereby inhibiting NIK activity (Supplementary Fig. 7aiii).

Our results showed LTβR-NFκB/RelA activation without IκB-degradation (Supplementary Fig. 7ai). Since TRAF2 and TRAF3 have been shown to be mediators^43^ or inhibitors^11,44^ of NFκB activation, respectively, the data implicate TRAF3 suppression of NFκB activity as predominant in resting LEC. TRAF3 may also suppress the recruitment of TRAF2 to the activated LTβR and prevent TRAF2-mediated classical NFκB signaling (Supplementary Fig. 7bii). Blockade of TRAF2 recruitment to LTβR by the ciLT decoy peptide also blocked TRAF3 binding to LTβR, prevented further TRAF2 and TRAF3 degradation induced by receptor activation (Supplementary Fig. 7biii), but did not affect LTβR-NIK activation. Overall, the results supported a model where TRAF2 was the mediator of classical NFκB signaling in LEC, and TRAF3 was the critical component for the LTβR-non-classical NFκB axis in LEC.

LTα1β2 is mainly expressed on activated lymphocytes and mature DCs^18,32–34,40^. LTβR is widely expressed on non-lymphoid endothelial cells including LEC and BEC, intestinal epithelial cells, and LN stromal cells^40^. The distribution of receptors and ligands suggests that the LTα1β2–LTβR axis serves as a bridge for immune cell trafficking between lymphoid and non-lymphoid tissues. NIK signaling primarily regulates this bridge as shown by blocking the signaling with nciLT, which suppressed CCL21 and CXCL12 production and prevented T cell and DC migration across LEC or lymphatic vessels. By expressing high levels of LTαβ, Treg preferentially and directly engaged LTβR-NIK signaling, compared to other CD4 T cells or DC. The Treg–LEC interaction resulted in enhanced TEM across LEC and triggered VCAM-1 and CCL21 expression by LEC. These data confirmed our previous report that LT signaling was particularly important for suppressive regulatory cell migration and LEC homeostasis^3^. Non-Treg CD4 T cells and DC, which expressed lower levels of LTα1β2, did not stimulate LEC LTβR but did require the constitutive LEC NIK activation for TEM. Presumably these CD4 T cells and DC migrate toward and across LEC via CCR7-CCL21 (ref.^45^). The differential use of the LTα1β2–LTβR axis by Treg versus non-Treg cells suggest that Treg may modify the migration of these other cells by modulating LEC responses. Our data show that nciLT inhibited naïve and activated T cell migration in vitro (Fig. 5a), and that nciLT inhibited naïve T cell migration in vivo (Figs. 5e, 6g). However, since it is generally considered that naïve T cells migrate via blood to LN, while activated and memory T cell migrate via tissues and lymph to LN^46^, future investigations should determine how the LT system regulates the migration of activated and memory CD4 and CD8 cells in vivo.

In vivo targeting of T cell and DC migration by the LTβR blocking peptides in CHS showed that these peptides and signaling pathways are relevant for disease; that individual kinetic components of immune responses, such as sensitization, elicitation, and resolution, can be precisely targeted; and that both lymphocytes and myeloid cells are equally influenced by these pathways. These findings open up the possibility for other applications and investigations of how lymphatic signaling and trafficking regulate immunity. These peptides may serve as a foundation for compound screening and drug discovery for novel therapeutics to regulate immune responses.

## Methods

### Mice

C57BL/6 (WT, LTβR^−/−^, *Ltα*^−/−^) (7–10 weeks) were purchased from The Jackson Laboratory (Bar Harbor, ME). Prox1-Cre-ER^T2^ mice were from Guillermo Oliver (St. Judes Hospital, Memphis, TN)^47^, and LTβR^fl/fl^ were from Thomas Hehlgans (University of Regensberg, Regensberg, Germany)^48^. For conditional depletion of LTβR in Prox1-expressing LEC and lymphatic vessels, Prox1-Cre-ER^T2+/−^LTβR^fl/fl^ (KO) and littermate control Prox1-Cre-ER^T2−/−^LTβR^fl/fl^ (WT) mice were injected i.p. with tamoxifen (Sigma-Aldrich, St. Louis, MO) 0.125 mg/g i.p suspended in corn oil for 5 consecutive days, followed by a chase period of 10 days. Foxp3GFP mice on a C57BL/6 background were from Dr. A. Rudensky (Memorial Sloan Kettering Cancer Center)^49^. All animal experiments were performed in accordance with Institutional Animal Care and Use Committee approved protocols.

### Cells

C57BL/6 mouse primary dermal LECs (C57-6064 L) were from Cell Biologics, Inc. (Chicago, IL), and were cultured according to the manufacturer’s instructions in manufacturer-provided mouse endothelial cell medium supplemented with 5% fetal bovine serum (FBS), 2 mM l-glutamine, 100 IU/mL penicillin, vascular endothelial growth factor, endothelial cell growth supplement, heparin, epidermal growth factor, and hydrocortisone. SVEC4-10 (CRL-2181) cells were from American Type Culture Collection, and were cultured in Dulbecco's modified Eagle's medium (DMEM) with 4.5 g/L glucose, containing 10% (v/v) FBS, 2 mM l-glutamine, 100 IU/mL penicillin, and 100 μg/mL streptomycin.

### Peptide synthesis and reconstitution

Peptides were synthesized by GenScript. All peptides were >95% purity, dissolved in endotoxin-free ultrapure water, and concentrations quantified by spectrophotometry^50^ after reconstitution and stored at −80 °C.

### CD4^+^ T cells and bone marrow derived dendritic cell

CD4^+^ T cells from mouse LNs and spleens were isolated using CD4^+^-negative selection (Stemcell Technologies, Cambridge, MA), and were cultured as previously described (1). Briefly CD4^+^CD25^−^Foxp3GFP^−^ WT or Ltα^−/−^ cells with >98% purity were sorted using a FACS Aria II (BD Biosciences). The sorted GFP^−^ naïve CD4 T cells (nCD4) (5 × 10^4^) were then co-cultured with T cell-depleted, 800 rad-irradiated C57BL/6 splenocytes as stimulator cells (5 × 10^4^) in U-bottom 96-well plates for 5 days at 37 °C in 5% CO_2_, with IL-2 (20 ng/mL; eBioscience, San Diego, CA), anti-CD3ɛ mAb (1 μg/mL, clone 145-2C11; eBioscience) for activated T cells (aCD4); and human TGF-β1 (10 ng/mL; eBioscience) for induced regulatory T cells (iTreg). Cells were cultured in RPMI 1640 supplemented with 10% FBS, 1 mM sodium pyruvate, 2 mM l-glutamine, 100 IU/mL penicillin, 100 μg /mL streptomycin, non-essential amino acids and 2 × 10^−5^ M 2-ME (Sigma-Aldrich). BMDCs were generated as described^51^. Briefly, bone marrow (BM) cells of wild-type mice were treated with 10 ng/mL GM-CSF (R&D Systems, Minneapolis, MN) for 10 days in Petri dishes, and the loosely attached cells were collected and CD11c^+^ DC were purified by CD11c-positive selection kit (Stemcell Technologies).

### Primary murine skin LEC purification and culture

Abdominal skin and ears from 5- to 6-week-old wild type and LTβR^−/−^ C57BL/6 mice were collected and digested in 4 mg/mL collagenase D (Roche, Indianapolis, IN) at 37 °C for 1 h. The dissociated cells were washed, depleted of CD45^+^ cells using MojoSort Mouse CD45 Nanobeads (Biolegend, Inc, San Diego, CA), re-suspended in mouse endothelial cell medium (Cell Biologics, Inc), and plated in six-well tissue culture plates for 3–5 days until the adherent cells reached confluency. The cells were dissociated with 0.25% trypsin-EDTA (Thermo Fisher, Waltham, MA) and the LEC were purified with Lyve-1-FITC (eBioscience) using anti-FITC positive selection kit (Stemcell Technologies).

### Flow cytometry

LECs in phosphate-buffered saline (PBS) containing 0.2 mM calcium, 0.1 mM magnesium, and 0.5% w/v bovine serum albumin (BSA) were treated with anti-CD16/32 (clone 93; eBioscience) to block Fc receptors, and then stained with antibodies to cell surface molecules in the same buffer. Flow cytometry antibody used was: APC-eflour780-anti-mouse CD4 (GK1.5; eBioscience); PE-anti-mouse CD25 (PC61.5; eBioscience); PE-anti-mouse CD44 (IM7; eBioscience); APC-anti-mouse VCAM-1 (clone 647; eBioscience). For detection of LTα2β1, T cells were incubated with MOPC21 or LTβRIg at 2 μg /mL for 60 min at 37 °C in HBSS with 2% FBS. Cells were washed, and then stained for 45 min at 4 °C with BV421-rat anti-mouse IgG1 along with antibodies to other cell surface markers. Cells were then washed and fixed with 4% paraformaldehyde and run on an LSR Fortessa flow cytometer (BD Biosciences, San Jose, CA). Results were analyzed with FlowJo 8.7 (Treestar). Flow cytometry gating information is included in Supplementary Fig 8.

### MTT viability assay

LECs were plated into 24-well tissue culture plates, incubated overnight, and treated with 20 μM peptides with or without agonist anti-LTβR (2 μg/mL) for 3 h. The cells were washed and followed by 3-h incubation with 0.5 mg/mL MTT (3-(4, 5-dimethylthiazol-2-yl)-2,5 diphenyl tetrazolium bromide) (Sigma-Aldrich). Fifty microliters DMSO was added to cells before reading OD at 540 nm.

### Evans Blue endothelial permeability assay

2.5 × 10^5^ primary LEC or SVEC4-10 were plated on inverted 0.2% (w/v) gelatin-coated Boyden chamber transwell polycarbonate membranes. The cell layers were treated with various conditions as noted in the text and figure legends prior to adding with 100 µL of 0.67 mg/mL Evans Blue (Sigma-Aldrich) diluted in IMDM migration medium containing fat-free BSA (40 mg/mL) (Gemini Bio-Products, Broderick, CA). Six hundred microliters fresh migration medium without phenol red was added to the lower chamber. After 0.5, 1, 2, and 3 h, lower chamber medium was collected into flat-bottom 96-well tissue culture (TC) plate, and the optical density at 650 nm was measured in a microplate reader (TECAN, San Jose, CA).

### T cell-LEC binding assay

2 × 10^4^ primary LECs or SVEC4-10 were plated on 0.2% (w/v) gelatin-coated flat-bottom 96-well TC plate. The LEC layers were treated with peptides or antibodies as noted in the text and figure legends before adding with 2 × 10^5^ CFSE-labelled CD4 T cells in IMDM migration buffer without a phenol red indicator. After 3 h at 37 °C in 5% CO_2_, non-bound cells were removed by gently washing and replacing with PBS containing 0.1 mM MgCl_2_ and CaCl_2_. Control wells with input CFSE-CD4 T cells remain unwashed. The fluorescein intensity (FI) from the bound CFSE-CD4 T cells in the plate was measured with a microplate reader. Percentage of bound CFSE-CD4 T cells were calculated as 100 × (FI of sample−FI of plate/cell background)/FI of input CFSE-CD4 T cells.

### Real-time PCR

One microgram of total RNA extracted using Trizol reagent (Invitrogen) was reverse-transcribed into cDNA with GoScript™ Reverse Transcription System (Promega, Fitchburg, WI). mRNA expression levels were quantified by real-time PCR using SYBR Green Master Mix with an ABI Prism 7900HT (Applied Biosystems, Foster City, CA). Values for specific gene expression were normalized to house-keeping HPRT gene expression, and were calculated as: 2^(Ct of HPRT−Ct specific gene). Sequences of the primer used were: mVCAM-1 forward 5′-GCAGGATGCCGGCATATACG-3′, mVCAM-1 reverse 5′-TGCGCAGTAGAGTGCAA GG A-3′; mCCL2 forward 5′-TCCACTACCTTTTCCACAACC-3′; mCCL2 Reverse: 5′-GGATCCACACCTTGCATTTAA-3′; mCXCL12 forward 5′-CTCTGCATCAGTGACGGTAA-3′, mCXCL12 reverse 5′-CTTCAGCCGTGCAACAATCT-3′; CCL21 forward 5′-AAGGCAGTGATGGAGGGG-3′ and CCL21 reverse 5′-CGGGTAAGAACAGGATTG-3′.

### ELISA

5 × 10^5^ LECs or SVEC4-10 cells were plated in 12-well plates and treated with 20 μM peptides for 30 min at 37 °C. Cells were then washed and incubated with anti-LTβR agonistic mAb (2 μg/mL) for 30 min at 4 °C, followed by washing and then crosslinking with anti-rat IgG (2 μg/mL) for 6 or 16 h at 37 °C. The supernatants were collected and stored in −80 °C. Mouse CCL2 and CCL21 were measured with ELISA kits from Biolegend, Inc. and R&D Systems, respectively.

### Immunoblotting and co-immunoprecipitation

LECs were lysed in buffer containing 20 mM Hepes (pH 7.4), 150 mM NaCl, 10 mM NaF, 2 mM Na_3_VO_4_, 1 mM EDTA, 1 mM EGTA, 0.5% Triton X-100, 0.1 mM DTT, 1 mM PMSF and protease inhibitor cocktail (Roche). Protein in the cell extract was quantified using protein quantification kit (Bio-Rad, Philadelphia, PA) and 10 μg total protein was run on Novex™ WedgeWell™ 4–20% Tris-Glycine Mini Gels (Invitrogen) and transferred to an Immobilon-P membrane (Bio-Rad). Membranes were probed with anti-p100/p52, NIK, phospho-IKKα/β (Ser176/180) (16A6), IκBα, TRAF2, TRAF3, and GAPDH antibodies. For co-IP assays, 500 μg total protein of cell extract was incubated with 1 μg of anti-mLTβR (5G11b) overnight, followed by 4 h incubation with 25 μL protein G Agarose beads (Sigma-Aldrich). The beads were then washed with lysis buffer and boiled in 2× Laemmli sample buffer (Bio-Rad). Uncropped immunoblotting images are included in Supplementary Fig 9.

### Transendothelial migration

Transmigrations across endothelial cells were described previously^3^. Briefly the inverted transwell insert (24-well; Corning International) with 5 μm pore size was coated with 0.2% (w/v) gelatin (Bio-Rad) for 1 h at 37 °C before loading with 1.5 × 10^5^ primary skin LEC or SVEC4-10 in 100 μL mLEC or cDMEM medium. After 2 days, the LEC cell layers were treated with various conditions as noted in the text and figure legends prior to adding 2 × 10^5^ T cells or DCs in 100 μL to the upper chamber of transwell plate while the lower chamber contained mCCL19 or CCL21 (50 ng/mL; R&D systems). All cells or reagents were prepared in IMDM containing transferrin and 0.5% (w/v) fatty acid-free BSA (Gemini). T cells that migrated to the lower chamber after 3 h at 37 °C were counted.

### Time-lapse microscopy

Purified naïve CD4 T cells were incubated with 5 μM CFSE at 37 °C for 5 min, quenched with 50% FBS at 4 °C for 5 min and were washed in migration buffer. CFSE-labelled CD4 T cells (5 × 10^4^ cells per transwell) migrating across endothelial monolayers to CCL19 (50 ng/ mL) were visualized by EVOS FL Auto Cell Imaging System (Thermo Fisher Scientific) with a ×20 objective. One image was captured every 5 min for 3 h. Cell tracks were analyzed with Volocity version 6.3 software (Perkin Elmer).

### Immunhistochemistry

LEC monolayers were stained for surface VCAM-1, LTβR, or Lyve-1 with rat anti-mouse VCAM-1 (Clone 429; eBioscience), rat anti-mouse LTβR (Clone 3C8; eBioscience), or rabbit-anti-mouse Lyve-1 (70R-LR003, Fitzgerald, Acton, MA), and then were fixed for 20 min at 4 °C with 4% (w/v) paraformaldehyde (Affymetrix). Cells were permeablized with PBS 0.2% (v/v) Triton X-100 (Sigma-Aldrich), and treated with 4% donkey serum for 30 min then incubated with rabbit-anti-mouse CXCL12 (eBioscience), goat-anti-mouse CCL21 (Clone AF457; R&D System), anti-NIK mouse monoclonal antibody (Clone A-12; Santa Cruz Biotechnology), or rabbit-anti-mouse RelA (Cell Signaling) or RelB (Thermo Fisher Scientific) for nuclear translocation staining overnight at 4 °C. The primary antibodies were detected with Alexa Fluor 448, 647 (Cy5), or 546 (Cy3)-conjugated donkey anti-goat, donkey anti-rabbit, or donkey anti-rat secondary antibodies (Jackson ImmunoResearch, West Grove, PA) for 1 h at 4 °C. Transwell membranes were transferred onto glass slides and visualized by fluorescent microscopy (Zeiss LSM 510 Meta and LSM5 Duo) with a ×60 or ×20 objective. Images were analyzed with Volocity version 6.3 software. For specific molecules, the fluorescence intensity was measured as mean fluorescence intensity over the entire field. For specific cells, the total number of cells stained with specific fluorescence was measured as total fluorescence intensity. Pearson’s correlation coefficient (PCC) was used to quantify the degree of co-localization between fluorophores.

### Footpad and whole-mount ear migration assays

Mice were anesthetized and 1 × 10^6^ CD25^+^ Tregs or CD4^+^ non-Tregs were injected intradermally into the footpads or ear pinnae in 20 μL PBS as we previously described. For footpad assays, draining popliteal LN were collected 12 h post paired injection (control vs nciLT or ciLT treatments) both footpads in each mouse and processed for flow cytometry. For ear pinnae assays, whole-mount ears were collected 4–16 h post injection, fixed for 10 min at room temperature with 4% paraformaldehyde, then stained with anti-Lyve-1 at 5 μg/ mL (70R-LR003, Fitzgerald), anti-CCL21 (AF457, R&D Systems) at 5 μg/mL in PBS, 0.5% BSA, and 0.3% Triton X-100 overnight at 4 °C; washed with PBS, 0.5% BSA, and 0.3% Triton X-100; stained with 5 μg/mL donkey anti-rabbit Alexa Fluor 488 and donkey anti-goat Cy3 (Jackson ImmunoResearch) for 2 h at 4 °C, washed twice in PBS, 0.5% BSA, and 0.3% Triton X-100; fixed once more as above, transferred to glass slides, and mounted with Prolong Gold (Life Technologies, Carlsbad, CA). Distance of T cells from lymphatic vessels calculated with minimum distance program in Volocity 6.3.

### CHS and FITC painting

Twenty-five microliters of 0.5% (v/v) DNFB in acetone/olive oil (4:1) solution was applied onto the shaved abdomen of the mice on days 0 and 1 (sensitization phase). On day 5, the right ear was treated with 20 μL 0.2% DNFB (10 μL on each side of the pinna), and the same for the left ear with 20 μL acetone/olive oil (challenge phase). Ear swelling was measured with a digimatic thickness gauge (Mitsutoyo, Kanagawa-ken, Japan) before and after 24 to 48 h DNFB challenge. The CHS response (ear swelling) was calculated by subtracting pre-challenge ear thickness for each animal. After euthanasia at the indicated days, the ears were removed and fixed with 10% formaldehyde in PBS and embedded in paraffin for hematoxylin and eosin staining, or were unfixed and embedded in OTC compound (Sakura Finetek, Torrance, CA), and processed for immunohistochemistry staining. In vivo DC migration was performed on shaved abdomen as described^52^. Briefly, fluorescein isothiocyanate (FITC, Sigma) 4 mg/mL was dissolved in 1:1 acetone:dibutyl phthalate (Sigma) and 50 µL applied to the abdominal skin. Recovered inguinal and axillary LNs were digested in 2 mg/mL collagenase D (Roche) for 30 min at 37 °C. Cells were then passed through a 100 µm cell strainer, and stained for flow cytometry.

### Statistical analysis

Numerical data are presented as mean ± SEM. Significance of differences was evaluated using one-way ANOVA and Prizm 5 software. Asterisks mark data statistically different from the controls, with *p*-values noted in the figure legends. A *p* value of <0.05 was considered significant for one-way ANOVA and Student *t*-tests. The number of replicates is noted in the figure legends.

### Data availability

The authors declare that [the/all other] data supporting the findings of this study are available within the paper and its supplementary information files.

## Electronic supplementary material

Supplementary Information
