# Author Correction: Regulation of T cell afferent lymphatic migration by targeting LTβR-mediated non-classical NFκB signaling

**DOI:** 10.1038/s41467-019-10952-0

**Published:** 2019-06-27

**Authors:** Wenji Piao, Yanbao Xiong, Konrad Famulski, C. Colin Brinkman, Lushen Li, Nicholas Toney, Chelsea Wagner, Vikas Saxena, Thomas Simon, Jonathan S. Bromberg

**Affiliations:** 1 Center for Vascular and Inflammatory Diseases, University of Maryland School of Medicine, Baltimore, MD 21201 USA; 2 Department of Surgery, University of Maryland School of Medicine, Baltimore, MD 21201 USA; 3 Department of Laboratory Medicine and Pathology, University of Alberta, 250 Heritage Medical Research Centre, Edmonton, AB T6G 2S2 Canada; 4 Department of Microbiology and Immunology, University of Maryland School of Medicine, Baltimore, MD 21201 USA

**Keywords:** Inflammatory diseases, Tumour-necrosis factors, Lymphatic vessels, NF-kappaB

Correction to: *Nature Communications* 10.1038/s41467-018-05412-0, published online 1 August 2018.

The original version of this Article contained an error in the fifth sentence of the ‘LTβR-derived peptides that target separate NFκB pathways’ section of the Results, which incorrectly read ‘ciLT (RQIKIWFQNRRMKWKKPEEGAPGP) included the (P/S/A/T)X(Q/E)E TRAF-binding motif required for TRAF2 but not TRAF3 binding to LTβR in the classical pathway^18,20^.’ The correct version states ‘RQIKIWFQNRRMKWKKTPEEGAPGP’ in place of ‘RQIKIWFQNRRMKWKKPEEGAPGP’—a ‘T’ has been added as the 17th character. This has been corrected in both the PDF and HTML versions of the Article.

