# Supplementary material for: Regulation of T cell afferent lymphatic migration by targeting LTβR-mediated non-classical NFκB signaling
Source: Nat Commun. 2018 Aug 1;9:3020. doi: 10.1038/s41467-018-05412-0 (PMC6070541; doi:10.1038/s41467-018-05412-0)
Supplement: Supplementary file 1 — Supplementary Information [file 41467_2018_5412_MOESM1_ESM.pdf]

Supplementary Information

Regulation of T cell afferent lymphatic migration by targeting LTβR-mediated non-classical NFκB signaling

Piao et al.

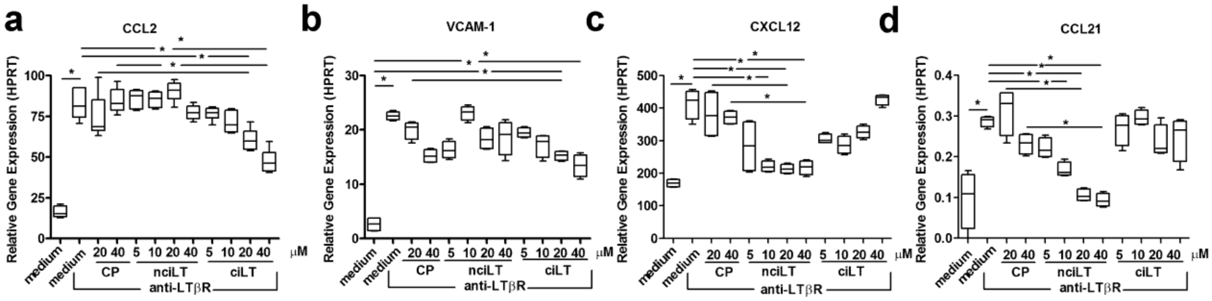

Supplementary Figure 1. *Dose-response for nciLT and ciLT.* qRT-PCR of LTβR-induced CCL2 (**a**), VCAM-1 (**b**), CXCL12 (**c**), and CCL21 (**d**) in SVEC4-10 treated with various concentrations (5-40 μM) of peptides. Cells lysed for total RNA extraction after 3-hour stimulation with 2 μg/mL anti-LTβR crosslinked with 2 μg/mL mouse anti-rat IgG1. Mean ± SEM of 2 independent experiments. *p < 0.05 by one-way ANOVA.

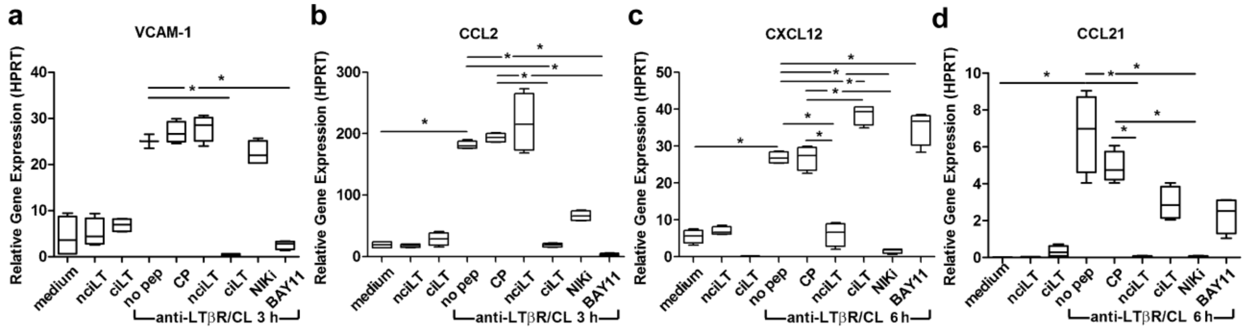

Supplementary Figure 2. *Classical and non-classical inhibitors differentially affect gene expression.* qRT-PCR of LTβR-induced VCAM-1 (**a**), CCL2(**b**), CXCL12 (**c**), and CCL21(**d**) in SVEC4-10 treated with indicated peptides (20 μM), NIKi (50 μM), or BAY11 (25 μM) and stimulated with 2 μg/mL anti-LTβR crosslinked with mouse anti-rat IgG1 (2 μg/mL). Mean ± SEM of 2 independent experiments. *$p < 0.05$ by one-way ANOVA.

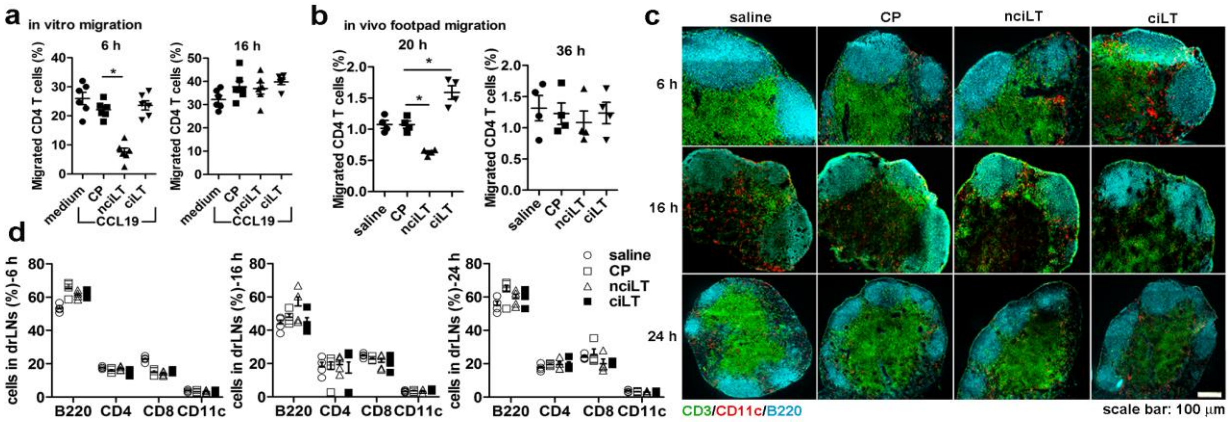

Supplementary Figure 3. *Kinetics of persistence of peptide effects in vitro and in vivo, and effects of peptide on draining LN architecture and cell content.* (**a**) LEC cell layers on inverted Boyden chamber treated with indicated peptides (20 μM) for 30 min, washed, and after 6 or 16 hours 2x10^5 purified naïve CD4 T cells loaded into the chamber and allowed to migrate for 3 hours toward 100 ng/mL CCL19. (**b**) Each hind footpad treated with 5 nmol of indicated peptides, after 20 or 36 hours, 1x10^6 CFSE-labelled naïve CD4 T cells injected into hind footpads. Local popliteal LNs collected and analyzed with flow cytometry 16 hours after cell transfer. (**c**) 5 nmol of indicated peptide injected into hind footpads. After 6, 16, and 24 hours, popliteal LNs collected and analyzed for T cell (CD3, CD4, and CD8), B cell (B220), and DC (CD11c) distribution by immunohistochemistry (**c**) and flow cytometry (**d**). Magnification 20x; scale bar 100 μm. Mean ± SEM of 2 independent experiments. *$p < 0.05$ by one-way ANOVA (**a** and **b**).

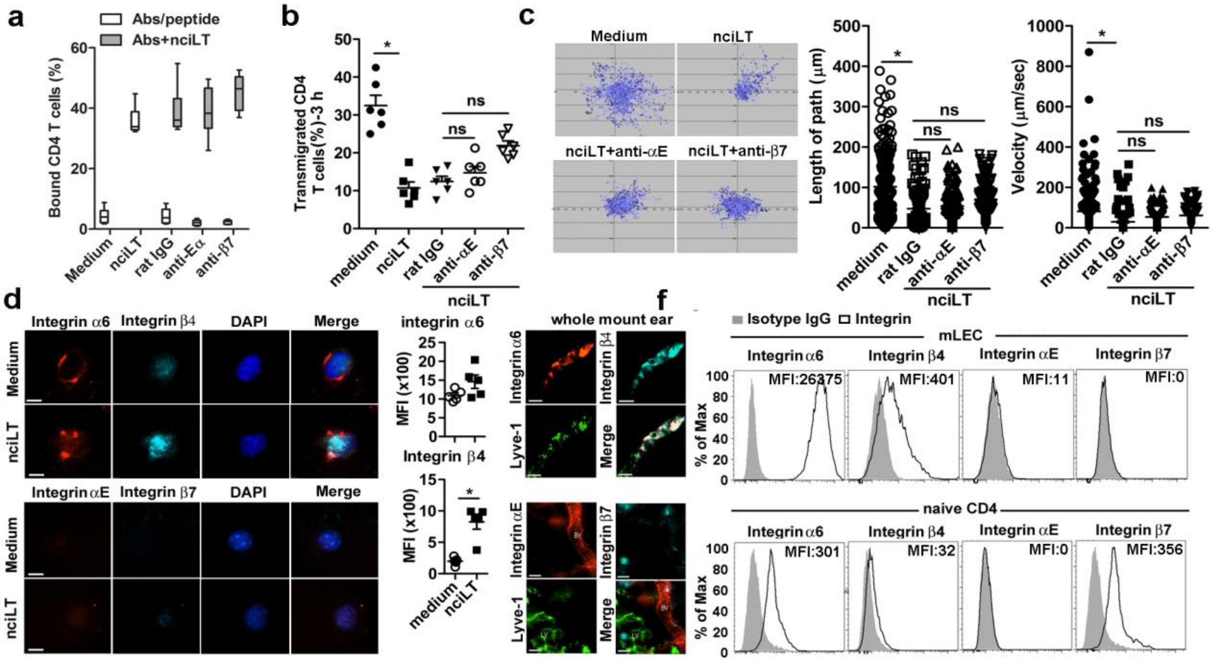

Supplementary Figure 4. *LEC and lymphatic vessels express integrins $\alpha 6$ and $\beta 4$*. (**a**) Binding of naïve CD4 T cells to LEC pretreated with nciLT (20 μM) with (filled bars) or without anti-integrin mAbs (white bars) (2 μg/ml) for 30 minutes at 37°C. (**b and c**) LEC layers treated as in **a**; naïve CD4 T cells migrated across LEC toward CCL19. Migrated cells in lower chamber counted (**b**) or monitored for 3 hours of live imaging for track length (left) and velocity (right) (**c**). (**d**) Immunohistochemistry of integrins on primary LEC pretreated with or without nciLT (20 μM) for 30 min. Magnification 60x; scale bar 10 μm. (**e**) Whole mount staining of wild type C57BL/6 ear pinnae for the indicated integrins. BV, blood vessel; LV, lymphatic vessel. Magnification 20x; scale bar 100 μm. (**f**) Flow cytometric analysis of integrin expression on primary LEC and purified naïve CD4 T cells. MFIs of positive cells shown. Data representative of 3 independent experiments. *$p < 0.05$ by one-way ANOVA (**a-d**).

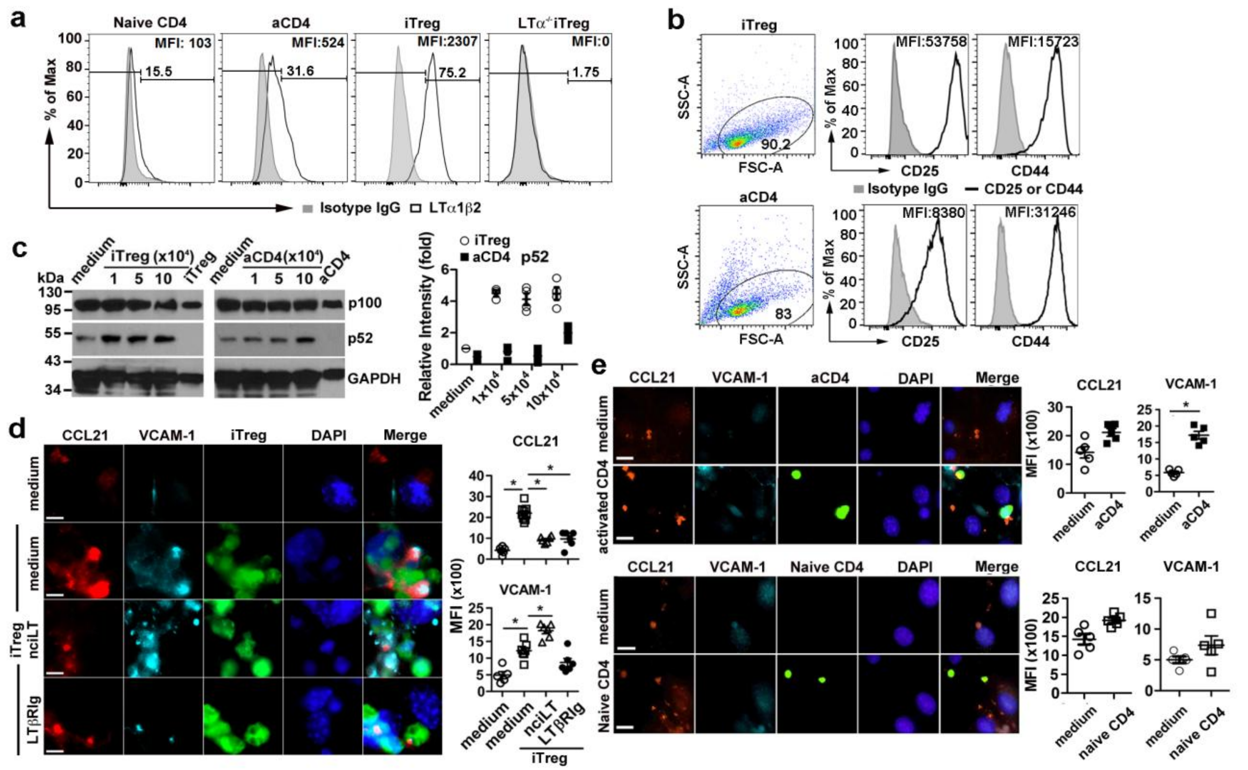

Supplementary Figure 5. *T lymphocytes engage LTαβ-LTβR NIK pathway and activate cell adhesion molecules and homing chemokines in LEC.* (**a and b**) Flow cytometric analysis for LTα1β2 expression on naïve CD4, activated CD4 (aCD4), iTreg, or LTα-deficient iTreg (**a**) and cell activation markers (CD25, CD44) on iTreg and aCD4 (**b**). Gating shows percent positive cells (**a**) or live cells (**b**) and MFI of positive cells. (**c**) Immunoblot of p100 to p52 conversion in LEC incubated with various numbers of purified iTreg or activated CD4 T cells for 6 hours. (**d-f**) Immunohistochemistry of VCAM-1 and CCL21 in primary LEC were pretreated with nciLT (20 μM) or LTβRIg (2 mg/mL) for 30 min at 37°C in 8-well slide chamber, and co-cultured with 2×10^{5} CFSE labeled iTreg (**d**), activated CD4 T cells (**e**), or naïve CD4 T cells (**f**) for 6 hours. Magnification 60x; scale bar 5 μm. *$p < 0.05$ by one-way ANOVA (**c, d**) or by Student's t test (**e, f**).

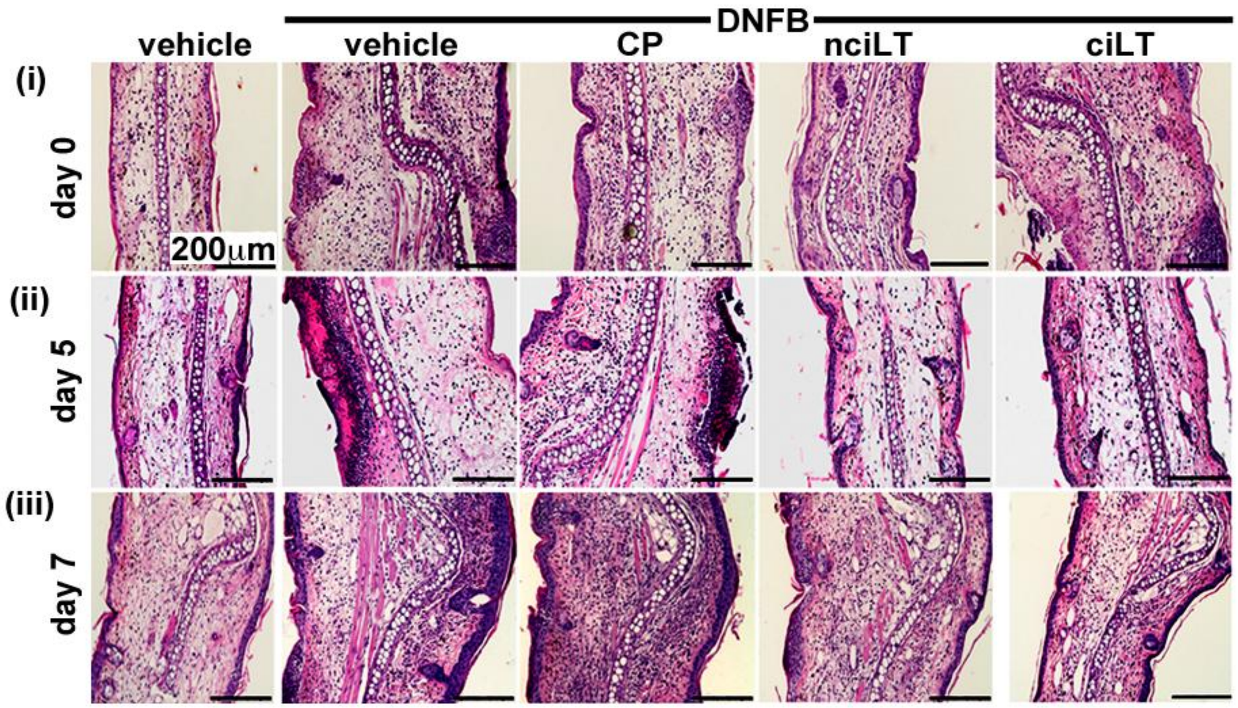

Supplementary Figure 6. *Histologic analysis of CHS.* H&E images of ear pinnae CHS responses of the indicated groups, treatments and times. Representative of three independent experiments with 3 mice/group for each experiment. Magnification 20x, scale bar 200 μm.

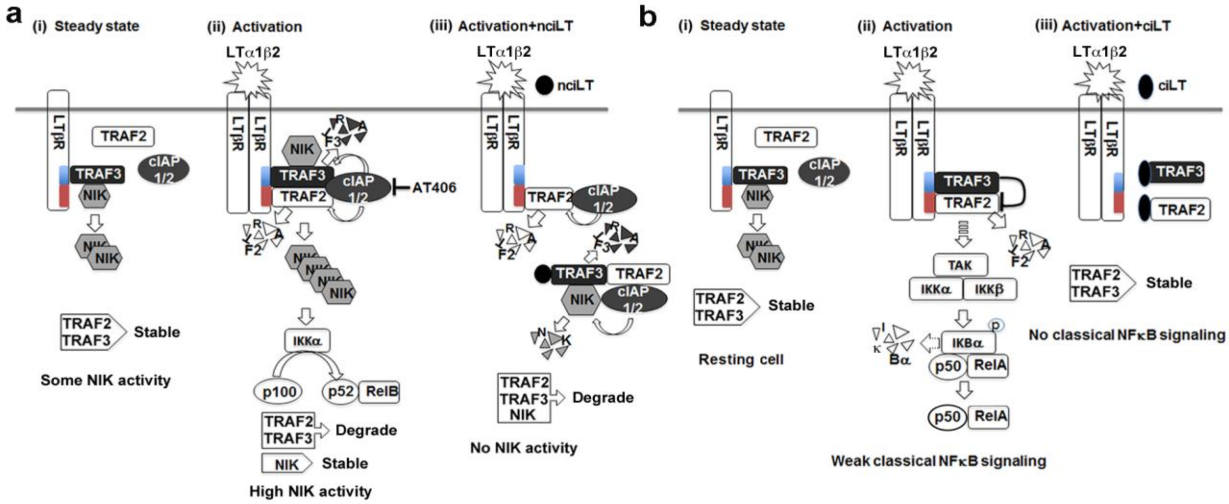

Supplementary Figure 7. *Proposed model of TRAF regulation by the blocking peptides in LTβR-mediated NFκB pathways.* (**a**) Non-classical NIK pathway predominates in LEC. (i) In steady state, TRAF3 constitutively binds LTβR and is stabilized from degradation (**Fig. 3b**, **3c**). (ii) LTβR activation recruits TRAF2 and cIAP1/2, which together with TRAF3 and NIK form a signal complex; both TRAF2 and TRAF3 are degraded by ubiquitination by cIAP1/2, leading to release of NIK from the complex. NIK activates IKKα which converts p100 to p52 (**Fig. 1f**). (iii) nciLT sequesters TRAF3 but not TRAF2 from the activated receptor, causing TRAF2 and TRAF3 degradation and preventing NIK activation (**Fig. 3b**, **3c**) and blocking RelB nuclear translocation (**Fig. 3g**). (**b**) (i) In steady state, the classical pathway is not activated by LTβR (**Fig. 1c, 1d, 3d, 3e**). (ii) With LTβR activation, TRAF2 recruits and activates TGFβ activating kinase 1 (TAK1) and the LTβR-classical NFκB pathway. TAK1 activates IKKα and IKKβ, which phosphorylate and degrade IκBα to release p50 and RelA into the nucleus (**Fig. 1d, 1e, 3e**). (iii) ciLT sequesters both TRAF2 and TRAF3 from LTβR (**Fig. 3b**), and stabilizes TRAF3 and inhibits TRAF2 activation to block RelA nuclear translocation (**Fig. 3c, 3e**).

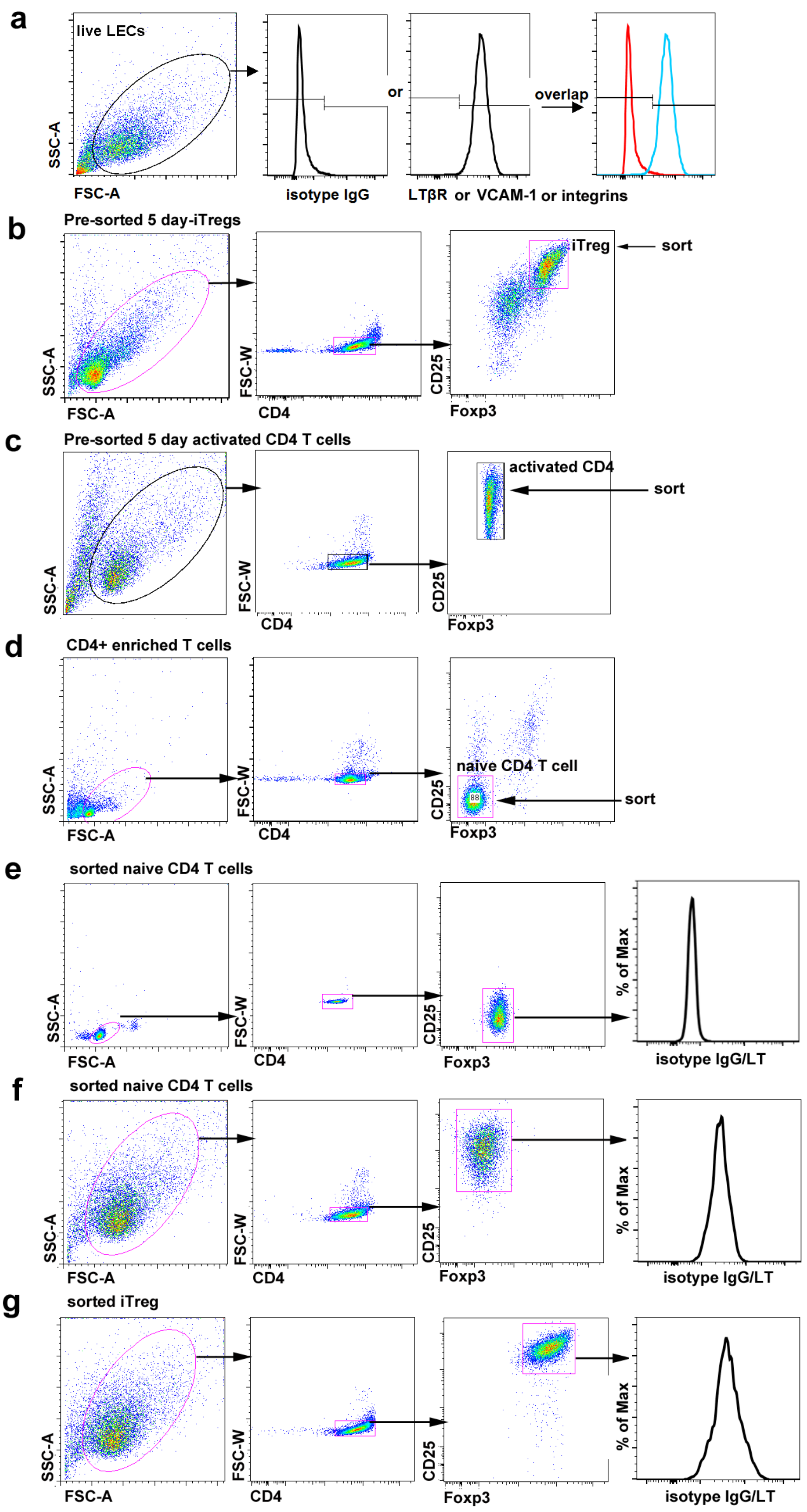

a
live LECs
SSC-A
FSC-A
isotype IgG
or
LTβR or VCAM-1 or integrins
overlap
b
Pre-sorted 5 day-iTregs
SSC-A
FSC-A
FSC-W
CD4
iTreg
sort
CD25
Foxp3
c
Pre-sorted 5 day activated CD4 T cells
SSC-A
FSC-W
CD4
activated CD4
sort
CD25
Foxp3
d
CD4+ enriched T cells
SSC-A
FSC-A
FSC-W
CD4
naive CD4 T cell
sort
CD25
Foxp3
e
sorted naive CD4 T cells
SSC-A
FSC-A
FSC-W
CD4
CD25
Foxp3
% of Max
isotype IgG/LT
f
sorted naive CD4 T cells
SSC-A
FSC-A
FSC-W
CD4
CD25
Foxp3
% of Max
isotype IgG/LT
g
sorted iTreg
SSC-A
FSC-A
FSC-W
CD4
CD25
Foxp3
% of Max
isotype IgG/LT

Supplementary Figure 8. *Gating strategies used for flow cytometry analysis or for cell sorting.* (**a**) Gating strategy to determine the percentage of LTβR, VCAM-1, or integrin expression on SVEC4-10 or mLEC in Fig. 1a (LTβR), 2b (VCAM-1), and S4f (integrins in mLEC or purified nCD4). (**b-d**) Gating strategy to sort Treg (CD4+CD25+Foxp3+), activated CD4 T (CD4+CD25+Foxp3-), and naïve CD4 T (CD4+CD25-Foxp3-) cells from Foxp3 reporter mice used on the in vitro migration assay presented in Fig. 5a, e-f or used in co-culture with LEC for immunoblot assay in Fig. 6i, j. (**e-g**) Gating strategy to determine the percentage of LTα1β2 expression on T cells presented in Fig. S5a. The same strategy was used for CD25 and CD44 expression on iTreg and aCD4 in Fig.S5b.

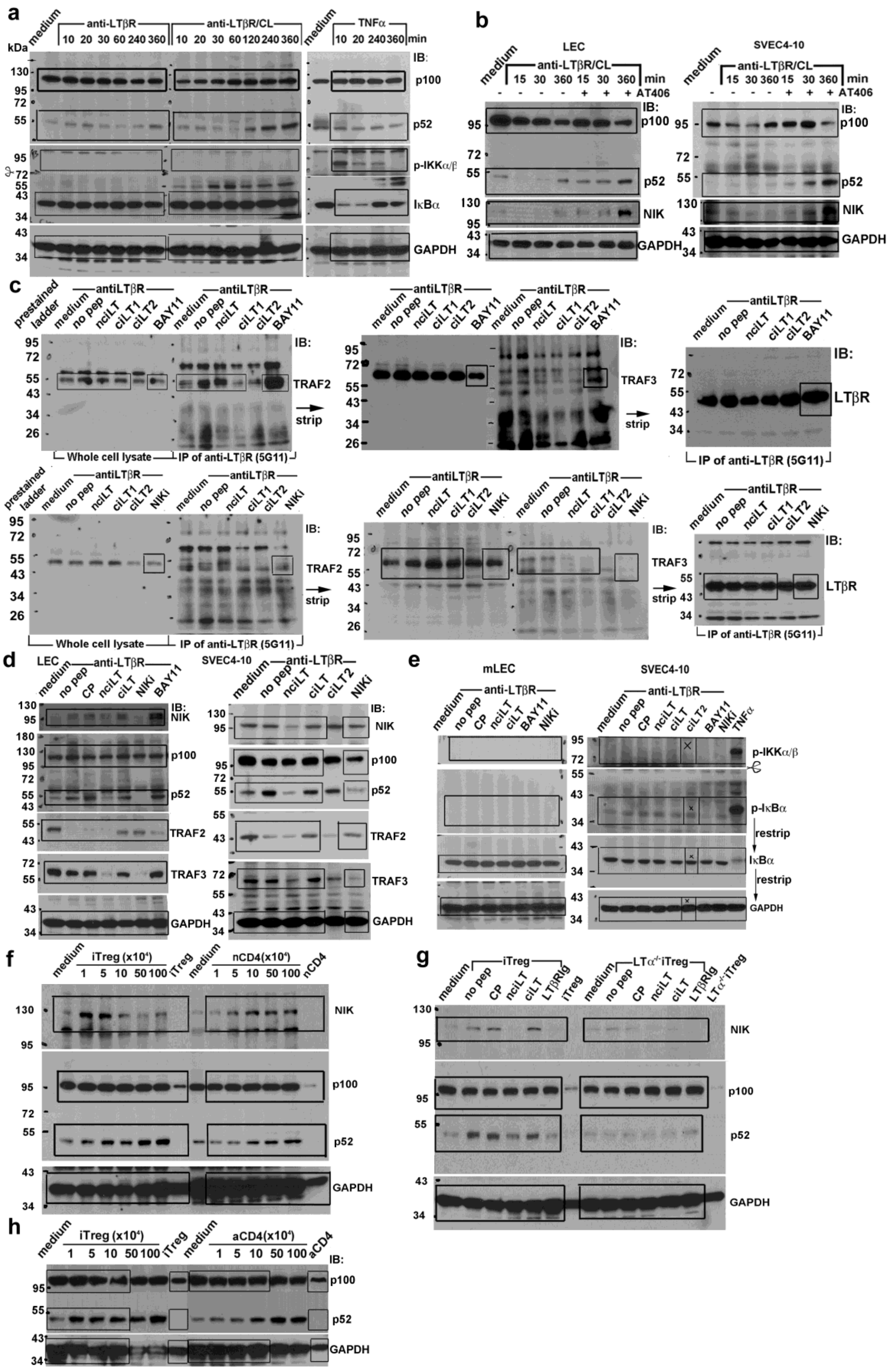
a
medium
anti-LTβR
10 20 30 60 240 360
anti-LTβR/CL
10 20 30 60 120 240 360
medium
TNFα
10 20 240 360 min
kDa
IB:
p100
p52
p-IKKα/β
IκBα
GAPDH
b
LEC
SVEC4-10
medium
anti-LTβR/CL
15 30 360 15 30 360 min
AT406
p100
p52
NIK
GAPDH
c
prestained ladder
antiLTβR
medium
no pep
nciLT
ciLT1
ciLT2
BAY11
NIKi
TRAF2
TRAF3
LTβR
strip
Whole cell lysate
IP of anti-LTβR (5G11)
d
LEC
SVEC4-10
anti-LTβR
CP
NIK
TRAF2
TRAF3
e
mLEC
SVEC4-10
anti-LTβR
p-IKKα/β
p-IκBα
restrip
IκBα
f
iTreg (x10⁴)
1 5 10 50 100
iTreg
nCD4(x10⁴)
nCD4
g
iTreg
LTα⁻/⁻iTreg
LTβRIg
h
aCD4(x10⁴)
aCD4

Supplementary Figure 9. Original Immunoblots. (**a**) Immunoblots corresponding to Fig. 1c. (**b**) Immunoblots corresponding to Fig. 1f. (**c**) Immunoblots corresponding to Fig. 3b. (**d**) Immunoblots corresponding to Fig. 3c. (**e**) Immunoblots corresponding to Fig. 3d. (**f**) Immunoblots corresponding to Fig. 6i. (**g**) Immunoblots corresponding to Fig. 6j. (**h**) Immunoblots corresponding to Fig. S5c.
